# The Dynamics of Viruslike Capsid Assembly and Disassembly

**DOI:** 10.1021/jacs.2c04074

**Published:** 2022-07-06

**Authors:** Suzanne B. P. E. Timmermans, Alireza Ramezani, Toni Montalvo, Mark Nguyen, Paul van der Schoot, Jan C. M. van Hest, Roya Zandi

**Affiliations:** † Bio-Organic Chemistry Research Group, Institute for Complex Molecular Systems, Eindhoven University of Technology, P.O. Box 513, 5600 MB Eindhoven, The Netherlands; ‡ Department of Physics and Astronomy, University of California, Riverside, California 92521, United States; § Soft Matter and Biological Physics Group, Department of Applied Physics, Eindhoven University of Technology, P.O. Box 513, 5600 MB Eindhoven, The Netherlands

## Abstract

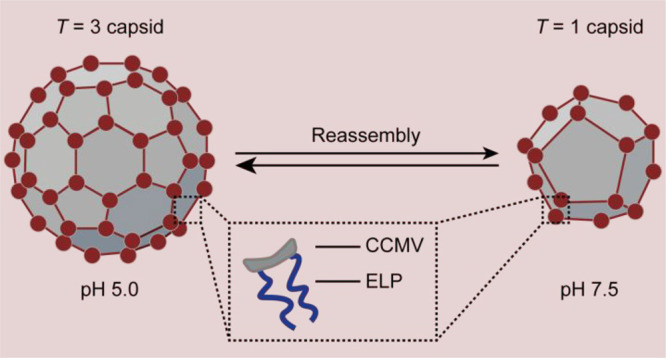

Cowpea chlorotic mottle virus (CCMV) is a widely used model for virus replication studies. A major challenge lies in distinguishing between the roles of the interaction between coat proteins and that between the coat proteins and the viral RNA in assembly and disassembly processes. Here, we report on the spontaneous and reversible size conversion of the empty capsids of a CCMV capsid protein functionalized with a hydrophobic elastin-like polypeptide which occurs following a pH jump. We monitor the concentrations of *T* = 3 and *T* = 1 capsids as a function of time and show that the time evolution of the conversion from one *T* number to another is not symmetric: The conversion from *T* = 1 to *T* = 3 is a factor of 10 slower than that of *T* = 3 to *T* = 1. We explain our experimental findings using a simple model based on classical nucleation theory applied to virus capsids, in which we account for the change in the free protein concentration, as the different types of shells assemble and disassemble by shedding or absorbing single protein subunits. As far as we are aware, this is the first study confirming that both the assembly and disassembly of viruslike shells can be explained through classical nucleation theory, reproducing quantitatively results from time-resolved experiments

Single-stranded RNA (ssRNA) viruses infect all species in the tree of evolution, causing significant economic damage and health concerns. The ssRNA genome of such viruses is protected by a shell called the capsid, composed of many copies of a single or a few protein subunits. To infect a host cell, a virus needs to enter, disassemble, release its genome, and use the cell’s machinery for replication. Clearly, the capsid is a responsive structure: Although it protects the genome and should be stable outside the cell, it must also readily disassemble once inside the cell and present its genome for replication.^1,2^

Arguably the most extensively studied viruses in this context are cowpea chlorotic mottle virus (CCMV) and Brome mosaic virus (BMV), which have proven to be good models for virus replication studies. The disassembly of the capsid in a cell must be triggered by changes in the chemical environment, resulting in the weakening of molecular interactions. Indeed, in vitro studies of CCMV and BMV show that following a pH jump from a neutral to a basic environment at high ionic strength the capsids of these viruses spontaneously disassemble.^3−5^ However, since the spatial and temporal resolution of intermediate structures of these studies are limited, kinetic pathways of disassembly have remained a mystery.

Generally, despite a huge body of work dedicated to understanding virus uncoating, our understanding of its kinetics and the factors contributing to it remains rudimentary.^6−15^ One of the main reasons for the lack of insight is the fact that the *assembly* of CCMV is governed by two driving forces involving two species, namely, the interaction between the capsid proteins (CPs) and that between the ssRNA and the RNA-binding domain of CPs.^16^ Distinguishing the contribution of both in the *disassembly* is not trivial, as CCMV shells in the absence of genome are not stable under physiological conditions.^17,18^

To develop and validate a plausible model that describes capsid assembly *and* disassembly, experimental conditions have to be found that allow for the elimination of the contribution of nucleic acids. This would not only lead to a better understanding of virus assembly but also allow for the development of tools to manipulate this process, either by preventing capsid formation and counteracting viral replication or by stabilizing empty capsids under physiological conditions as tools for diagnostic and therapeutic applications.^19^

Several years ago we designed the CP variant ELP-CP, which involves the attachment of elastin-like polypeptides (ELPs) at the N-terminus of the CPs of CCMV.^20^ These ELPs consist of nine repeating Val-Pro-Gly-Xaa-Gly pentapeptide units, which switch from an extended water-soluble state to a collapsed hydrophobic state in response to an increase in temperature and/or electrolyte concentration.^21^ The sequence contains 2 times the Trp, 2 times the Val, 4 times the Leu, and 1 time Gly as the guest residues (Xaa). At pH 5, the ELP-CPs form viruslike particles (VLPs) with a diameter of 28 nm, similar to the native *T* = 3 particles.^20^ At pH 7.5, wild-type CPs do not assemble into shells, yet ELP-CPs assemble into 18 nm (*T* = 1) VLPs upon increasing the salt concentration, a process induced by the hydrophobicity of the ELPs.^20,22,23^

In this paper, we describe the results from time-resolved experiments, allowing us to investigate the disassembly of one type of ELP-CP capsid and reassembly of another in response to pH changes (Figure 1). While changing the pH from 5 to 7.5, we monitor as a function of time how the *T* = 3 shells disappear, while the *T* = 1 shells appear. We also study the disassembly of *T* = 1 capsids and the assembly of *T* = 3 capsids by lowering the pH from 7.5 to 5. Our experimental findings can be explained by a simple model based on classical nucleation theory (CNT) applied to viruslike capsids,^24−27^ accounting for the time-evolution of the concentrations of the various species that result from the shedding or addition of single protein subunits as the different types of shell assemble and disassemble. As far as we are aware, this is the first study confirming that both assembly and disassembly of viruslike shells can be explained through CNT as a possible mechanism for quantitatively reproducing experimental data.

**Figure 1:**
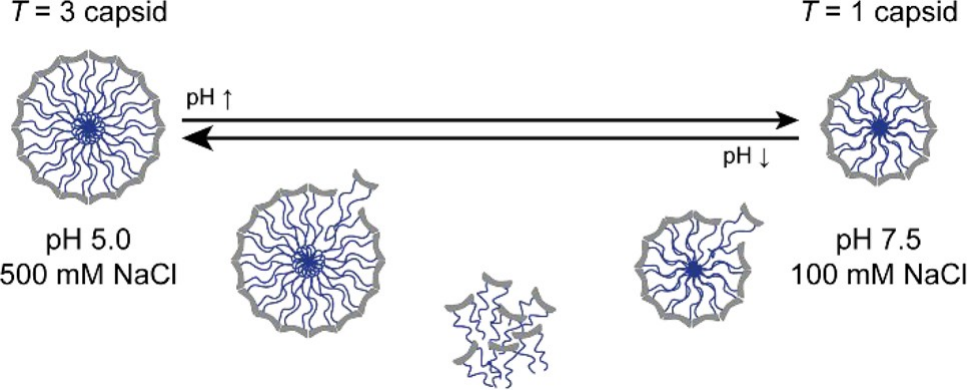
Schematic overview of the size change of ELP-CP viruslike particles (VLPs) upon a shift in pH.

For this purpose, we investigate the T number conversion over time, using a combination of size exclusion chromatography (SEC) and transmission electron microscopy (TEM). We first evaluate the conversion dynamics from *T* = 3 to *T* = 1 particles. Hereto, we dialyzed a 100 μM solution of empty VW1-VW8 ELP-CCMV *T* = 3 capsids at 4 °C from a pH 5.0 buffer with 500 mM NaCl to a pH 7.5 buffer with 100 mM NaCl, thus simultaneously increasing the pH and decreasing the ionic strength of the buffer environment. In order to stabilize the samples during SEC measurements, 0.2 equiv of Ni^2+^ was added (Supporting Information section 2.3 for optimization). Details of our experimental procedures are found in Supporting Information sections 1 and 2. As our experiments reveal that this process is very much dependent on the NaCl concentration in the buffer (Figures S1 and S2), we conclude that it must be driven by the stimulus-responsive ELP-domains. Because of this NaCl dependency, we changed the NaCl concentration from 500 mM at pH 5.0, to ensure stable *T* = 3 capsids, to 100 mM at pH 7.5, to reduce the strength of ELP-interactions and to ensure dynamics. Monitoring the capsid assembly state (Figure 2A,B) shows that after a short lag time, on the same time scale needed for the equilibration of the NaCl concentration during dialysis (Figure S2B), the shift from *T* = 3 to *T* = 1 capsids takes place via a rapid initial process, followed by one that is more gradual (Figure 2B). The complete capsid size transition takes months. Further evaluation with TEM (Figure 2E) confirms this transition process. To get a better understanding of the mechanism of transition, we performed experiments in which we added fluorescently labeled ELP-CPs to unlabeled capsids. We observe that at both pH 5.0 and 7.5 the capsids can exchange dimers with the solution (Figures S11 and S12), which makes it plausible that the observed size change involves the transfer of dimers. Furthermore, we note that it is unlikely for one structure to morph into the other one without disassembly because of the change in the radius of curvature between the two structures. If the sizes of the two structures were close to each other, then it would be possible for the big pieces of one shell to be recycled to form another shell.^28^

**Figure 2:**
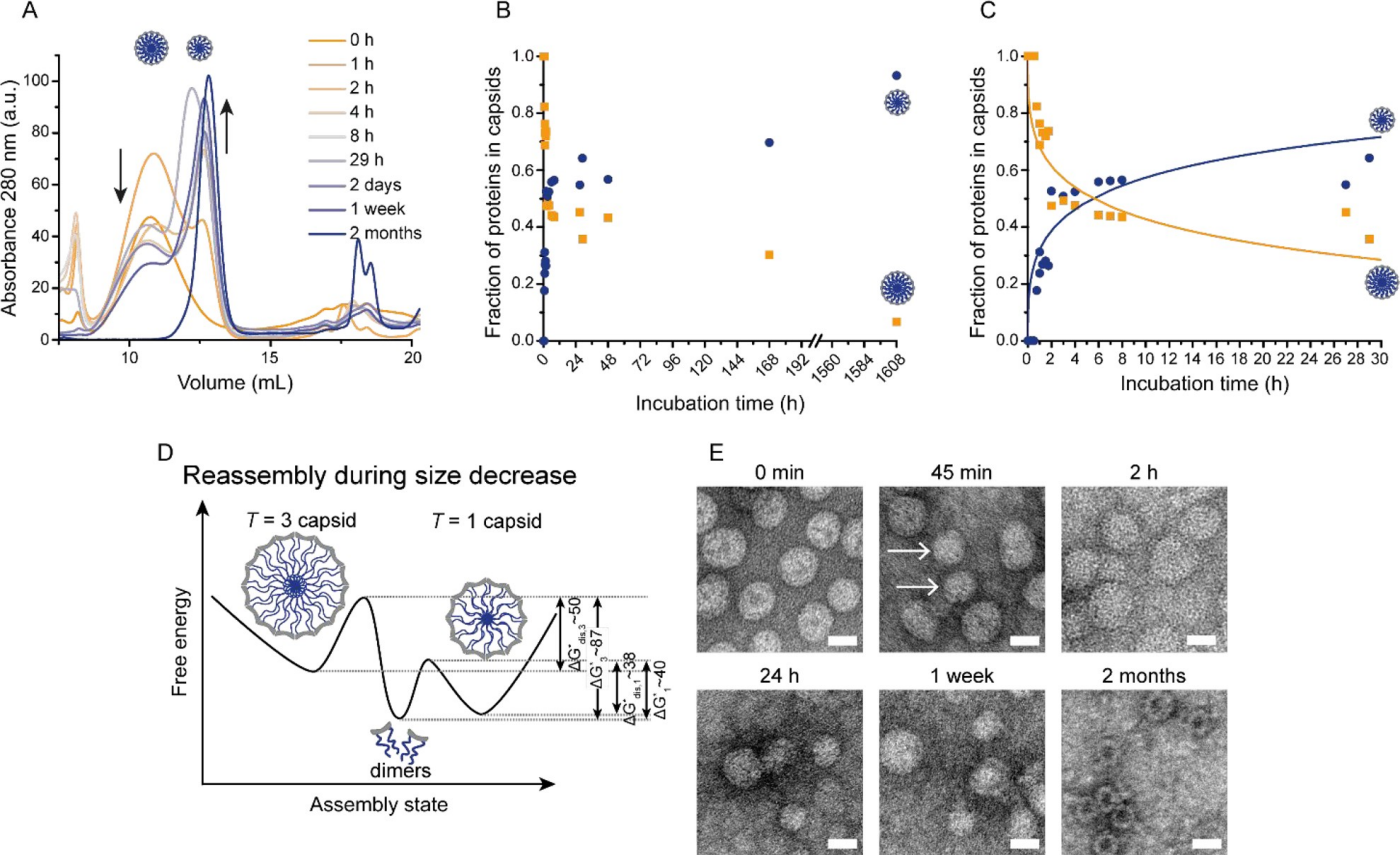
Analysis of ELP-CCMV capsids during the transition from *T* = 3 to *T* = 1 particles at pH 7.5. (A) SEC chromatograms measured after indicated dialysis times to pH 7.5. (B and C) Protein fractions as *T* = 1 (blue circles) and *T* = 3 (yellow squares) capsids as determined by integration of the SEC chromatograms (see also Figures S7–S9). The solid lines are the results of our numerical solution (eqs 3 and 4). See Table S4 for more details. (D) Schematic overview of the proposed reassembly mechanism during size decrease, where *T* = 1 capsids are energetically most favorable under the buffer conditions used. Δ*G* values are in *k*_B_*T* units. Energy barriers are not drawn to scale; the values provided are indicative. (E) TEM micrographs of samples that were taken after the indicated dialysis times. *T* = 1 capsids in the 45 min image are indicated with arrows. Scale bars correspond to 20 nm. Overview images and additional time points are depicted in Figure S10.

Our experiments suggest that we are pitting the assembly rate of one species against the disassembly rate of another. In order to explore the role of metastability in our experiments, we resort to CNT, as a plausible model to describe the system. Within CNT, the steady-state capsid assembly and disassembly rates *J*_as,*T*_ and *J*_dis,*T*_ can be written as^26,29^
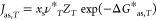
1
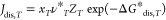
2where ν_T_*, *Z*_*T*_, *x*_s_, and *x*_*T*_ denote the attempt frequency of dimers attaching to the critical nucleus, the Zeldovich factor, and the mole fraction of free subunits, and the capsid of a given *T* number (Supporting Information section 3.1). Δ*G*_as,T_^*^ is the height of energy barrier between the free proteins and fully formed capsids, while Δ*G*_dis,T_^*^ is the height of the free energy barrier between the assembled and free CPs (Figures 2D and 3D for the opposite size shift). The barrier height depends on the overall protein concentration and on the binding free energies of the proteins in the two types of shell, *g*_*T*_, in units of thermal energy, averaged over all subunits of a fully formed capsid. The kinetic equations describing the concentration of dimers and *T* = 1 and *T* = 3 capsids read as
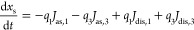
3and
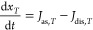
4where *q*_1_ and *q*_3_ are the numbers of dimers in fully formed *T* = 1 and 3 capsids, respectively. The quantities on the left-hand sides of eqs 3 and 4 represent time derivatives of the concentrations of the species in our model. The terms on the right-hand sides are due to the formation or dissociation of capsids. We solve the above system of equations numerically, using an explicit forward Euler method with adaptive time steps (Supporting Information section 3.2).

**Figure 3:**
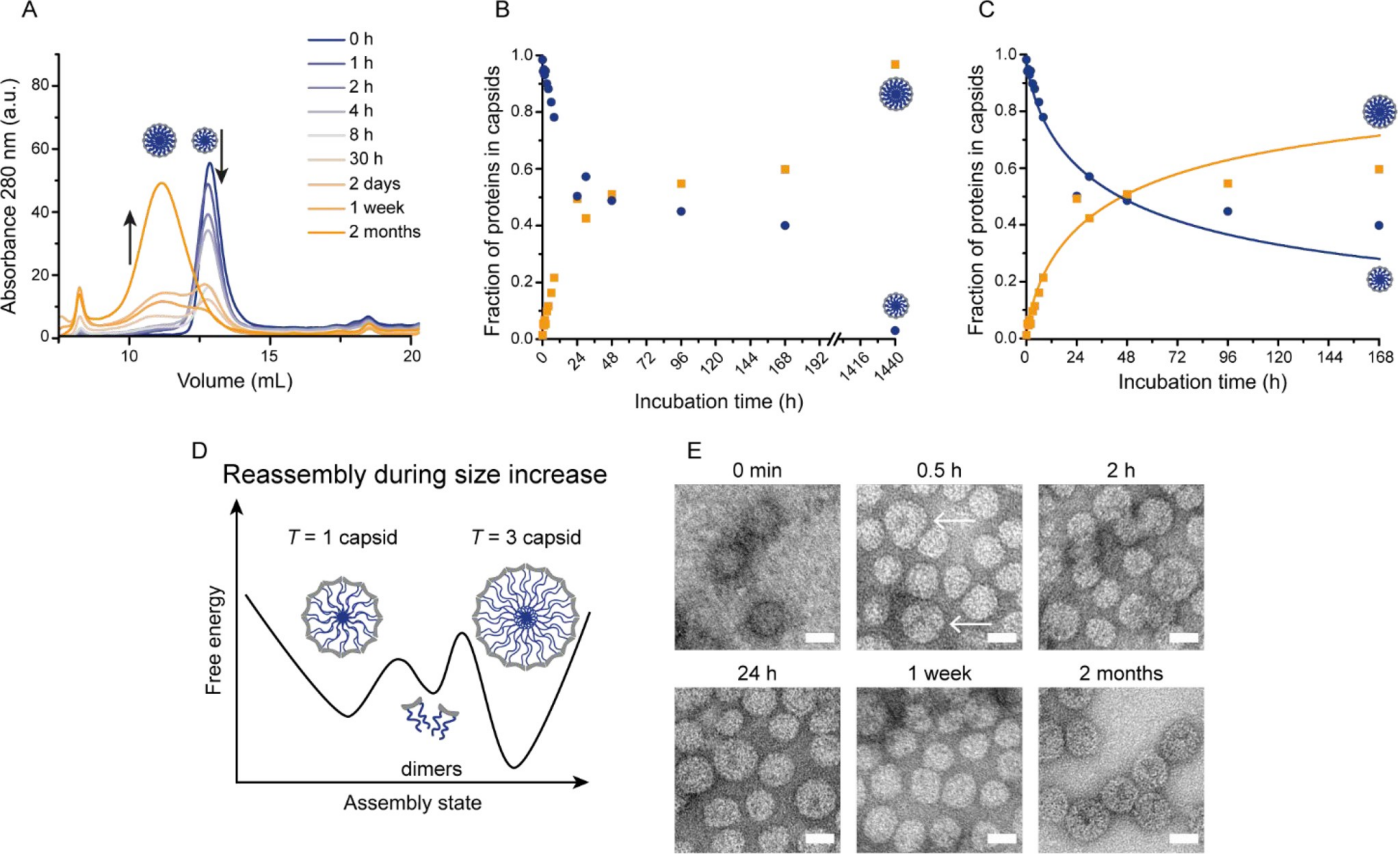
Analysis of ELP-CCMV capsids during transition from *T* = 1 to *T* = 3 particles at pH 5.0. (A) SEC chromatograms measured after indicated dialysis times to pH 5.0. (B and C) Protein fractions as *T* = 1 (blue circles) and *T* = 3 (yellow squares) capsids as determined by integration of the SEC chromatograms (see also Figures S13–S15. The solid lines are the results of our numerical solution (eqs 3 and 4). See Table S5 for more details. (D) Schematic overview of the proposed reassembly mechanism during size increase, where *T* = 3 capsids are energetically most favorable under the buffer conditions used. Δ*G* values are in *k*_B_*T* units. Energy barriers are not drawn to scale; the values provided are indicative. (E) TEM micrographs of samples taken after the indicated dialysis times. The *T* = 3 capsids in the 0.5 h image are indicated with arrows. Scale bars correspond to 20 nm. Overview images and additional time points are depicted in Figure S16.

Consistent with the experiments (Figure 2B), we find that upon increasing the pH from 5 to 7.5, the amount of *T* = 3 structures decreases while at the same time the number of *T* = 1 structures increases, indicating that under these experimental conditions the protein–protein attraction is stronger between subunits forming *T* = 1 shells than that of those forming *T* = 3 ones. Our curve fits in Figure 2C for times up to 30 h give *g*_1_ = −15.0 and *g*_3_ = −14.7 in thermal energy units (Supporting Information section 3.1).

As the *T* = 3 shells disassemble, the concentration of free dimers increases and, at some point, reaches the value of the critical capsid concentration *c*_1_^*^ = *e*^g_1_^, whereupon *T* = 1 shells start forming and consuming free dimers. As the free dimer concentration continues to increase, the disassembly rate of the *T* = 3 shells decreases, and the assembly rate of *T* = 1 shells increases, explaining the behavior of the disassembly and assembly curves shown in Figure 2C. However, fairly quickly the free dimer concentration attains a more or less constant value because the disassembly of *T* = 3 shells produces dimers that are immediately depleted by the formation of *T* = 1 shells, confirming that the changes in protein fraction in the capsids are due to the disassembly of *T* = 3 and assembly of *T* = 1 (Supporting Information section 3.3). We note that the decrease in free dimer concentration after two months in Figure 2A could be due to the fact that dimer proteins at pH 7.5 after prolonged storage are not highly stable and some aggregation and denaturation will occur over time. The theory presented in this paper does not include this effect.

We next discuss the size shift from *T* = 1 to *T* = 3 following a jump in pH from 7.5 to 5 at a constant NaCl concentration of 500 mM. Herein, a 100 μM solution of empty VW1-VW8 ELP-CCMV *T* = 1 capsids in a pH 7.5 buffer with 500 mM NaCl was dialyzed to a pH 5.0 buffer with 500 mM NaCl at 4 °C, during which the capsid assembly state was monitored with SEC and TEM measurements. Figure 3A,B shows that *T* = 1 particles, stable at neutral pH, disappear over time, while *T* = 3 particles appear. The whole process proceeds much more gradually than the opposite size shift and takes around 2 months to reach full completion (Figure 3B). We follow the dynamics with TEM (Figure 3E), confirming the increase in the number of *T* = 3 particles.

The number of *T* = 1 structures decreases and the amount of *T* = 3 structures increases in parallel, which points at stronger attractive interactions between CPs in the native species at low pH. Our curve fits in Figure 3C for times up to 168 h give *g*_1_ = −15.0 and *g*_3_ = −15.4 in thermal energy units. Again we find that the free subunit concentration very quickly becomes more or less constant: The disassembly of *T* = 1 shells produces dimers that are used for the formation of *T* = 3 shells.

From Figures 2B,C and 3B,C, it appears that *T* = 3 capsids easily dissociate at pH 7.5, crossing the growing fraction of *T* = 1 capsids after 6 h, while the disassembly of *T* = 1 CPs at pH 5.0 is much slower, crossing the growing fraction of *T* = 3 capsids only after 48 h. This is expected because the smaller size of a *T* = 1 capsid produces fewer subunits per disassembled shell. ELPs are positioned closer next to each other because of the higher curvature of *T* = 1 shells, and the interaction between ELPs remains strong at pH 5.0.

In this context we note that under certain conditions the association and dissociation of empty capsids is characterized by hysteresis: It is easier for capsids to assemble than to disassemble.^30^ Hence, assembled capsids can be significantly more stable kinetically than they are thermodynamically, implying that the height of the free energy barrier must be larger for disassembly than it is for assembly.^26,31^ For the experiments described in this paper, this means that the disassembly step must be rate-limiting if the unstable shells are to be converted into stable shells of a different size. This is indeed what we also find from our theoretical calculations.

In conclusion, we find that ELP-CPs can reversibly switch between *T* = 1 and 3 structures upon changing the solution conditions. While we have not ruled out the possibility that other models can also describe our experiments, remarkably, the interconversion between the two structures can be quite accurately described at least for initial and intermediate times by CNT. At pH 7.5, the driving force for the assembly of coat proteins is the interaction between the ELPs, while at pH 5.0 the attractive interaction between capsid proteins predominates over the attractive ELP–ELP interactions. Since ELPs are attached to the capsid proteins, the ELP-CCMVs do form a shell at pH 7.5, but only the smallest possible one as the ELPs need to be as close as possible to each other to make contact. This insight is of importance not only for a more fundamental understanding of virus assembly but also for the improved design of VLP-based nanomedicines.

## Supporting Information Available

Experimental methods for protein expression and analysis of self-assembly dynamics, supplemental discussions regarding the method optimizations, theoretical methods and discussions, and additional SEC chromatograms and TEM micrographs (PDF)

## Abbreviations

BMV: Brome mosaic virus
CCMV: cowpea chlorotic mottle virus
CNT: classical nucleation theory
CP: coat protein
ELP: elastin-like polypeptide
ss: single-stranded
SEC: size-exclusion chromatography
TEM: transmission electron microscopy
VLP: viruslike particle

## References

[ref1] Bruinsma R. F.; Wuite G. J. L.; Roos W. H. Physics of Viral Dynamics. Nat. Rev. Phys. 2021, 3, 76–91. 10.1038/s42254-020-00267-1. 33728406 PMC7802615

[ref2] Hagan M. F. Modeling Viral Capsid Assembly. Adv. Chem. Phys. 2014, 155, 1–68. 10.1002/9781118755815.ch01. 25663722 PMC4318123

[ref3] Bancroft J. B.; Hiebert E. Formation of an Infectious Nucleoprotein from Protein and Nucleic Acid Isolated from a Small Spherical Virus. Virology 1967, 32 (2), 354–356. 10.1016/0042-6822(67)90284-X. 6025882

[ref4] Bancroft J. B. The Self-Assembly of Spherical Plant Viruses. Adv. Virus Res. 1970, 16, 99–134. 10.1016/S0065-3527(08)60022-6. 4924992

[ref5] Adolph K. W.; Butler P. J. Studies on the Assembly of a Spherical Plant Virus. I. States of Aggregation of the Isolated Protein. J. Mol. Biol. 1974, 88 (2), 327–341. 10.1016/0022-2836(74)90485-9. 4452998

[ref6] Chevreuil M.; Lecoq L.; Wang S.; Gargowitsch L.; Nhiri N.; Jacquet E.; Zinn T.; Fieulaine S.; Bressanelli S.; Tresset G. Nonsymmetrical Dynamics of the HBV Capsid Assembly and Disassembly Evidenced by Their Transient Species. J. Phys. Chem. B 2020, 124 (45), 9987–9995. 10.1021/acs.jpcb.0c05024. 33135897

[ref7] Zhou J.; Zlotnick A.; Jacobson S. C. Disassembly of Single Virus Capsids Monitored in Real Time with Multicycle Resistive-Pulse Sensing. Anal. Chem. 2022, 94 (2), 985–992. 10.1021/acs.analchem.1c03855. 34932317 PMC8784147

[ref8] Yamauchi Y.; Greber U. F. Principles of Virus Uncoating: Cues and the Snooker Ball. Traffic Cph. Den. 2016, 17 (6), 569–592. 10.1111/tra.12387. PMC7169695 26875443

[ref9] Michaels T. C. T.; Bellaiche M. M. J.; Hagan M. F.; Knowles T. P. J. Kinetic Constraints on Self-Assembly into Closed Supramolecular Structures. Sci. Rep. 2017, 7 (1), 12295 10.1038/s41598-017-12528-8. 28947758 PMC5613031

[ref10] Hagan M. F.; Chandler D. Dynamic Pathways for Viral Capsid Assembly. Biophys. J. 2006, 91 (1), 42–54. 10.1529/biophysj.105.076851. 16565055 PMC1479078

[ref11] Keef T.; Micheletti C.; Twarock R. Master Equation Approach to the Assembly of Viral Capsids. J. Theor. Biol. 2006, 242 (3), 713–721. 10.1016/j.jtbi.2006.04.023. 16782135

[ref12] Harms Z. D.; Selzer L.; Zlotnick A.; Jacobson S. C. Monitoring Assembly of Virus Capsids with Nanofluidic Devices. ACS Nano 2015, 9 (9), 9087–9096. 10.1021/acsnano.5b03231. 26266555 PMC4753561

[ref13] Moerman P.; van der Schoot P.; Kegel W. Kinetics versus Thermodynamics in Virus Capsid Polymorphism. J. Phys. Chem. B 2016, 120 (26), 6003–6009. 10.1021/acs.jpcb.6b01953. 27027925

[ref14] Zlotnick A. To Build a Virus Capsid: An Equilibrium Model of the Self Assembly of Polyhedral Protein Complexes. J. Mol. Biol. 1994, 241 (1), 59–67. 10.1006/jmbi.1994.1473. 8051707

[ref15] Rapaport D. C. Role of Reversibility in Viral Capsid Growth: A Paradigm for Self-Assembly. Phys. Rev. Lett. 2008, 101 (18), 186101 10.1103/PhysRevLett.101.186101. 18999841

[ref16] Zandi R.; van der Schoot P. Size Regulation of Ss-RNA Viruses. Biophys. J. 2009, 96 (1), 9–20. 10.1529/biophysj.108.137489. 18931258 PMC2710049

[ref17] Garmann R. F.; Comas-Garcia M.; Knobler C. M.; Gelbart W. M. Physical Principles in the Self-Assembly of a Simple Spherical Virus. Acc. Chem. Res. 2016, 49 (1), 48–55. 10.1021/acs.accounts.5b00350. 26653769

[ref18] Zandi R.; Dragnea B.; Travesset A.; Podgornik R. On Virus Growth and Form. Phys. Rep. 2020, 847, 1–102. 10.1016/j.physrep.2019.12.005.

[ref19] Sun X.; Cui Z. Virus-Like Particles as Theranostic Platforms. Adv. Ther. 2020, 3 (5), 1900194 10.1002/adtp.201900194.

[ref20] van Eldijk M. B.; Wang J. C.-Y.; Minten I. J.; Li C.; Zlotnick A.; Nolte R. J. M.; Cornelissen J. J. L. M.; van Hest J. C. M. Designing Two Self-Assembly Mechanisms into One Viral Capsid Protein. J. Am. Chem. Soc. 2012, 134 (45), 18506–18509. 10.1021/ja308132z. 23101937 PMC3510441

[ref21] Urry D. W. Physical Chemistry of Biological Free Energy Transduction As Demonstrated by Elastic Protein-Based Polymers. J. Phys. Chem. B 1997, 101 (51), 11007–11028. 10.1021/jp972167t.

[ref22] Timmermans S. B. P. E.; Vervoort D. F. M.; Schoonen L.; Nolte R. J. M.; van Hest J. C. M. Self-Assembly and Stabilization of Hybrid Cowpea Chlorotic Mottle Virus Particles under Nearly Physiological Conditions. Chem. - Asian J. 2018, 13 (22), 3518–3525. 10.1002/asia.201800842. 29975459

[ref23] Schoonen L.; Maas R. J. M.; Nolte R. J. M.; van Hest J. C. M. Expansion of the Assembly of Cowpea Chlorotic Mottle Virus towards Non-Native and Physiological Conditions. Tetrahedron 2017, 73 (33), 4968–4971. 10.1016/j.tet.2017.04.038.

[ref24] Panahandeh S.; Li S.; Marichal L.; Leite Rubim R.; Tresset G.; Zandi R. How a Virus Circumvents Energy Barriers to Form Symmetric Shells. ACS Nano 2020, 14 (3), 3170–3180. 10.1021/acsnano.9b08354. 32115940

[ref25] Panahandeh S.; Li S.; Zandi R. The Equilibrium Structure of Self-Assembled Protein Nano-Cages. Nanoscale 2018, 10 (48), 22802–22809. 10.1039/C8NR07202G. 30516220

[ref26] Zandi R.; van der Schoot P.; Reguera D.; Kegel W.; Reiss H. Classical Nucleation Theory of Virus Capsids. Biophys. J. 2006, 90 (6), 1939–1948. 10.1529/biophysj.105.072975. 16387781 PMC1386774

[ref27] Kashchiev D. Kinetics of Nucleation. In Nucleation; Elsevier, 2000; pp 113–290.

[ref28] Panahandeh S.; Li S.; Dragnea B.; Zandi R. Virus Assembly Pathways Inside a Host Cell. ACS Nano 2022, 16 (1), 317–327. 10.1021/acsnano.1c06335. 35019271

[ref29] Luque Santolaria A. Structure, Mechanical Properties, and Self-Assembly of Viral Capsids. Doctoral Thesis. Universitat de Barcelona, 2011 (in Spanish).

[ref30] Singh S.; Zlotnick A. Observed Hysteresis of Virus Capsid Disassembly Is Implicit in Kinetic Models of Assembly. J. Biol. Chem. 2003, 278 (20), 18249–18255. 10.1074/jbc.M211408200. 12639968

[ref31] van der Schoot P.; Zandi R. Kinetic Theory of Virus Capsid Assembly. Phys. Biol. 2007, 4 (4), 296–304. 10.1088/1478-3975/4/4/006. 18185007

