# Supplementary material for: The Dynamics of Viruslike Capsid Assembly and Disassembly
Source: J Am Chem Soc. 2022 Jul 6;144(28):12608–12. doi: 10.1021/jacs.2c04074 (PMC9305980; doi:10.1021/jacs.2c04074)
Supplement: Supplementary file 1 — ja2c04074_si_001.pdf [file ja2c04074_si_001.pdf]

Supporting Information

The dynamics of virus-like capsid assembly and disassembly

Suzanne B. P. E. Timmermans,[1‡] Alireza Ramezani,[2‡] Toni Montalvo,[2] Mark Nguyen,[2] Paul van der Schoot,[3] Jan C. M. van Hest,[1*] Roya Zandi[2*]

1 Bio-Organic Chemistry Research Group, Institute for Complex Molecular Systems, Eindhoven University of Technology, P.O. Box 513, 5600 MB Eindhoven, The Netherlands; 2 Department of Physics and Astronomy, University of California Riverside, California 92521, USA; 3 Soft Matter and Biological Physics Group, Department of Applied Physics, Eindhoven University of Technology, P.O. Box 513, 5600 MB Eindhoven, The Netherlands.

[‡] These authors contributed equally to this work.

Contents

1 Experimental materials and methods S2

1.1 Materials S2

1.2 Buffers S2

1.3 UV-vis absorbance measurements S2

1.4 Mass spectrometry S2

1.5 Size exclusion chromatography (SEC) S3

1.6 Transmission electron microscopy (TEM) S3

1.7 Dynamic light scattering (DLS) measurements S3

1.8 Conductivity measurements S3

1.9 Native PAGE S4

1.10 General protocol for the expression of His_6-ELP-CCMV variants S4

1.11 General protocol for measuring the self-assembly dynamics during size reduction (conversion dynamics from T=3 to T=1 particles) S6

1.12 General protocol for measuring the self-assembly dynamics during size increase (conversion dynamics from T=1 to T=3 particles) S6

1.13 General protocol for mEGFP-labeling of capsids by exchange with labeled dimers S6

2 Experimental supplemental discussions S7

2.1 Optimization of conditions to allow for optimal dynamics S7

2.2 Optimization of dialysis conditions S9

2.3 Optimization of the SEC protocol for studying the self-assembly dynamics during size reduction S10

3 Theoretical methods and discussions S12

3.1 Theory S12

3.2 Numerics S17

3.3 The fraction of protein dimers in free solution and in capsids S18

3.4 Fitting of the simulation results with respect to different reference points S19

3.5 Table of parameters S20

4 Supplemental figures S22

5 References S29

1 Experimental materials and methods

1.1 Materials

Ampicillin, chloramphenicol, yeast extract, and peptone were purchased from Sigma-Aldrich/ Merck. Isopropyl-β-D-thiogalactopyranoside (IPTG) was obtained from PanReac AppliChem VWR. Ni-NTA agarose beads were obtained from Qiagen.

1.2 Buffers

Table S1 – Composition of buffers

Name	Composition
pH 5.0 buffer	50 mM NaOAc, 500 mM NaCl, 10 mM $MgCl_2$, 1 mM EDTA, pH 5.0
pH 5.0 no EDTA buffer	50 mM NaOAc, 500 mM NaCl, 10 mM $MgCl_2$, pH 5.0
pH 7.5 100 mM NaCl buffer	50 mM Tris·HCl, 100 mM NaCl, 10 mM $MgCl_2$, 1 mM EDTA, pH 7.5
pH 7.5 100 mM NaCl no EDTA buffer	50 mM Tris·HCl, 100 mM NaCl, 10 mM $MgCl_2$, pH 7.5
pH 7.5 500 mM NaCl buffer	50 mM Tris·HCl, 500 mM NaCl, 10 mM $MgCl_2$, 1 mM EDTA, pH 7.5
4x native PAGE loading buffer	248 mM Tris·HCl, 40 % glycerol (*v*/*v*), 0.02 % bromophenol blue (*w*/*v*)
Native PAGE running buffer	25 mM Tris·HCl, 192 mM glycine

All buffers were filtered over a 0.2 micron filter prior to use.

1.3 UV-vis absorbance measurements

In order to determine the protein concentrations during experiments, the absorbance at 280 nm was measured with a spectrophotometer ND-1000 and the concentrations were subsequently calculated using the theoretical extinction coefficients.[1]

1.4 Mass spectrometry

Protein mass characterization was performed using a High Resolution LC-MS system consisting of a Waters ACQUITY UPLC I-Class system coupled to a Xevo G2 Quadrupole Time of Flight (Q-ToF). The system consisted of a Binary Solvent Manager and a Sample Manager with Fixed-Loop (SM-FL). Proteins were separated (0.3 mL/min) on the column (Polaris C18A reverse phase

column, 2.0 x 100 mm, Agilent) using an acetonitrile gradient in water (15% to 75%, *v*/*v*) supplemented with formic acid (0.1%, *v*/*v*) before analysis in positive mode in the mass spectrometer. Deconvoluted mass spectra were obtained using the MaxENT1 algorithm in the Masslynx v4.1 (SCN862) software. Isotopically averaged molecular weights were calculated using the 'Protein Calculator v3.4' at http://protcalc.sourceforge.net. Protein samples were desalted by spin-filtration with MilliQ prior to measurement.

1.5 Size exclusion chromatography (SEC)

SEC analysis was performed on a Superose 6 increase 10/300 GL column (GE Healthcare Life Sciences). Analytical measurements were executed on an Agilent 1260 bio-inert HPLC. Samples with a protein concentration of 100 µM were separated on the column at 21 °C with a flow rate of 0.5 mL/min. Running buffer was "pH 7.5, 100 mM NaCl no EDTA buffer" for T = 3 to T = 1 shift and "pH 5.0 buffer" for T = 1 to T = 3 shift (see table S1 for exact compositions).

1.6 Transmission electron microscopy (TEM)

TEM grids (FCF-200-Cu, EMS) were glow-discharged using a Cressington 206 carbon coater and power unit. Protein samples (10 µM, 5 µL) were applied on the glow-discharged grids and incubated for 1 min. The samples were carefully removed using a filter paper. Then, the grid was negatively stained by applying 2% uranyl acetate in water (5 µL). The staining solution was removed after 15 seconds and the grid was allowed to dry for at least 15 minutes. The samples were studied on a FEI Tecnai 20 (type Sphera) (operated at 200 kV, equipped with a LaB_6 filament and a FEI BM-Ceta CCD camera).

1.7 Dynamic light scattering (DLS) measurements

DLS measurements were performed on a Malvern Zetasizer Nano ZSP at 21°C, unless stated otherwise. Samples (100 µM, unless stated otherwise) were centrifuged twice prior to analysis. Buffers were filtered prior to use. All measurements were done in triplo, and the average of the triplo measurements was plotted.

1.8 Conductivity measurements

Conductivity measurements were performed using a Mettler Toledo SevenGo Duo Pro pH/ conductivity meter SG78 that was calibrated with a 1413 µS/cm @ 25 °C standard (VWR, 84135.260). In order to assess the NaCl concentration during dialysis, a standard curve was prepared by measuring the conductivity of a series of pH 7.5 buffers with NaCl concentrations

ranging from 0 M to 1 M (with 100 mM intervals). As such, the following formula was used: $C(mS/cm) = 0.076 \times [NaCl](mM) + 6.1536$ with $R^2 = 0.9991$.

1.9 Native PAGE

Samples were prepared by mixing 7.5 µL of a 100 µM protein solution and 2.5 µL of 4x native PAGE loading buffer. 2.67 µL of the samples were loaded on a 4-20 % gel (Bio-Rad, Mini-PROTEAN TGX Precast Gels cat# 456-1096). The gel was run at 4 °C with pre-cooled native PAGE running buffer containing a cooling pack for 3 hours at 100 V. The gel was washed for 5 minutes in demineralized water and then stained with coomassie blue staining solution (Bio- Rad, cat# 161-0786). Subsequently, the gel was destained in demineralized water and visualized with an ImageQuant 350 gel imager with ImageQuant 350 Capture software.

1.10 General protocol for the expression of His_6-ELP-CCMV variants

The pET-15b-His_6-ELP-CCMV(ΔN26), pET-15b-His_6-VW1-VW8 ELP-CCMV(ΔN26) and pET15b-mEGFP-His_6-VW1-VW8 ELP-CCMV(ΔN26) vectors encoding for the three ELP-CCMV variants used in this manuscript were previously constructed.[2,3]
The native ELP-CCMV, VW1-VW8 ELP-CCMV and mEGFP-VW1-VW8 ELP-CCMV capsid protein variants were expressed according to standard expression procedures.[2,4,5] As an example, *E.coli* BLR(DE3)pLysS glycerol stocks containing the pET-15b-H_6-VW1-VW8-ELP-CCMV(ΔN26) vector were used for an overnight culture at 37 °C in LB medium (50 mL), containing ampicillin (100 mg/L) and chloramphenicol (50 mg/L). The overnight culture was used to inoculate 2xTY medium (1 L), containing ampicillin (100 mg/L) and grown at 37 °C till an optical density (OD_{600}) between 0.4 and 0.6 was reached. Protein expression was then induced by addition of IPTG (1 mM) and the culture was incubated at 30°C for 6 hours. Cells were harvested by centrifugation (2700 x *g*, 15 min, and 4 °C) and pellets were stored overnight at -20 °C.
The cell pellet was resuspended in lysis buffer (50 mM NaH_2PO_4, 300 mM NaCl, 10 mM imidazole, pH 8.0; 25 mL). Cell lysis was performed by ultrasonic disruption (7-10 times 30 seconds, 70% amplitude, Branson Sonifier 150). Cell lysate was then centrifuged (16.000 x *g*, 15 min, 4 °C) to remove bacterial debris. The supernatant was incubated with Ni-NTA agarose beads (3 mL) for 1 hour at 4 °C, followed by column loading. The flow-through was collected and the column was washed twice with wash buffer (50 mM NaH_2PO_4, 300 mM NaCl, 20 mM imidazole, pH 8.0; 20 mL). The proteins of interest were eluted from the column with elution buffer (50 mM NaH_2PO_4, 300 mM NaCl, 250 mM imidazole, pH 8.0; 1 time 0.5 mL, 7 times 1.5 mL) and fractions containing histidine-tagged VW1-VW8 ELP-CCMV were combined and dialyzed against pH 7.5 dimer buffer

(50 mM Trizma base, 500 mM NaCl, 10 mM $MgCl_2$, 1 mM EDTA, pH 7.5; 3 times 30-60 minutes using a 12-14 kDa tubing). The protein was then dialyzed against pH 5.0 capsid buffer (50 mM Trizma base, 500 mM NaCl, 10 mM $MgCl_2$, 1 mM EDTA, pH 5.0; 2 times 30-60 minutes, followed by overnight dialysis using a 12-14 kDa tubing) for stable storage at 4°C. The purity and characteristics of the protein were verified and determined by SDS-PAGE, SEC, Q-TOF mass spectrometry, DLS and TEM. The protein yields after purification and the Q-TOF results are listed in Table S2. The amino acid sequences of the three ELP-CCMV variants are listed in Table S3.

Table S2 – Expression yields and Q-TOF results of ELP-CCMV variants

Name	Yield (mg/L)	Q-TOF results	
		Calculated MW (Da)	Observed MW (Da)
Native ELP-CCMV	10-48	22253.4	22253.4
VW1-VW8 ELP-CCMV	22-34	22427.6	22427.2
mEGFP-VW1-VW8 ELP-CCMV	15-22	49807.5	49807.4

Table S3 – Amino Acid sequences of ELP-CCMV variants

Name	Sequence
Native ELP-CCMV	GHHHHHHVPGVGVPGLGVPGVGVPGLGVPGVGVPGLGVPGGGVPGVGVPGLGLEVVQPVIVEPIASGQGKAIKAWTGYSVSKWTASCAAAEAKVTSAITISLPNELSSERNKQLKVGRVLLWLGLLPSVSGTVKSCVTETQTTAAASFQVALAVADNSKDVVAAMYPEAFKGITLEQLTADLTIYLYSSAALTEGDVIVHLEVEHVRPTFDDSFTPVY
VW1-VW8 ELP-CCMV	GHHHHHHVPG**W**GVPGLGVPGVGVPGLGVPGVGVPGLGVPGGGVPG**W**GVPGLGLEVVQPVIVEPIASGQGKAIKAWTGYSVSKWTASCAAAEAKVTSAITISLPNELSSERNKQLKVGRVLLWLGLLPSVSGTVKSCVTETQTTAAASFQVALAVADNSKDVVAAMYPEAFKGITLEQLTADLTIYLYSSAALTEGDVIVHLEVEHVRPTFDDSFTPVY
mEGFP-VW1-VW8 ELP-CCMV	MVSKGEELFTGVVPILVELDGDVNGHKFSVSGEGEGDATYGKLTLKFICTTGKLPVPWPTLVTTLTYGVQCFSRYPDHMKQHDFFKSAMPEGYVQERTIFFKDDGNYKTRAEVKFEGDTLVNRIELKGIDFKEDGNILGHKLEYNYNSHNVYIMADKQKNGIKVNFKIRHNIEDGSVQLADHYQQNTPIGDGPVLLPDNHYLSTQSKLSKDPNEKRDHMVLLEFVTAAGITLGMDELYKGSGSMGHHHHHHVPG**W**GVPGLGVPGVGVPGLGVPGVGVPGLGVPGGGVPG**W**GVPGLGLEVVQPVIVEPIASGQGKAIKAWTGYSVSKWTASCAAAEAKVTSAITISLPNELSSERNKQLKVGRVLLWLGLLPSVSGTVKSCVTETQTTAAASFQVALAVADNSKDVVAAMYPEAFKGITLEQLTADLTIYLYSSAALTEGDVIVHLEVEHVRPTFDDSFTPVY

1.11 General protocol for measuring the self-assembly dynamics during size reduction (conversion dynamics from T=3 to T=1 particles)

For a typical dynamics experiment a 100 µM VW1-VW8 ELP-CCMV coat protein solution (150 µL – 1200 µL) in pH 5.0 buffer was prepared and dialyzed to “pH 5.0 no EDTA” buffer at 4 °C o.n. (12-14 kDa MWCO). Dialysis buffer (150 mL – 200 mL) was changed after 30 minutes and 60 minutes. Subsequently, the protein solution was dialyzed to “pH 7.5, 100 mM NaCl no EDTA” buffer at 4 °C (12-14 kDa MWCO) with dialysis buffer (150 mL – 200 mL) changes after 30 minutes and 60 minutes. At different time points during dialysis, 110 µL samples were retrieved from the dialysis membrane and incubated with 0.2 eq. of $NiCl_2$ (20 µM) for 50 minutes at room temperature. Subsequently, samples were spun down for 1 minute at 13400 rpm and subjected to SEC analysis. In addition, 5 µL samples were retrieved from the dialysis membrane, spun down for 1 minute at 13400 rpm, and subjected to TEM analysis.

1.12 General protocol for measuring the self-assembly dynamics during size increase (conversion dynamics from T=1 to T=3 particles)

For a typical dynamics experiment a 100 µM VW1-VW8 ELP-CCMV coat protein solution (150 µL – 1200 µL) in pH 7.5 buffer with 500 mM NaCl was prepared and dialyzed to pH 5.0 buffer at 4 °C (12-14 kDa MWCO). Dialysis buffer (150 mL – 200 mL) was changed after 30 minutes and 60 minutes. At different time points during dialysis, 110 µL and/or 5 µL samples were retrieved from the dialysis membrane, spun down for 1 minute at 13400 rpm, and subjected to SEC analysis and/ or TEM analysis respectively.

1.13 General protocol for mEGFP-labeling of capsids by exchange with labeled dimers

For a typical dynamics-based mEGFP-labeling experiment both VW1-VW8 ELP-CCMV and mEGFP-VW1-VW8 ELP-CCMV protein solutions were dialyzed overnight at 4 °C to pH 7.5 buffer with 100 mM NaCl (12-14 kDa MWCO). Subsequently, the protein solutions were mixed in a 4:1 ratio (VW1-VW8 ELP-CCMV : mEGFP-VW1-VW8 ELP-CCMV) and incubated at 4 °C for 4 hours. Hereafter, the mixtures were dialyzed at 4 °C either to pH 5.0 buffer (*T*=3 capsids) or to pH 7.5 buffer, 500 mM NaCl (*T*=1 capsids) and incubated in the final buffer at 4 °C for up to one week.

At intermediate time points, samples were taken, heated to 21 °C with 1 °C/min and subjected to SEC analysis with pH 5.0 buffer or pH 7.5 buffer with500 mM NaCl as eluent. At the final time point, fractions were collected during a preparative SEC run and the combined capsid fractions were analyzed with SDS-PAGE and TEM. The amount of mEGFP incorporated into the capsids was determined by SDS-PAGE analysis. Gels that were visualized via Coomassie Brilliant Blue staining (Biorad) were analyzed with ImageJ gel analysis software to calculate the loading of capsids with mEGPF. Hereto, the following formula was used:

$$labelling\ efficiency = \frac{gel_{GFP-ELP-CCMV} / mw_{GFP-ELP-CCMV}}{gel_{ELP-CCMV} / mw_{ELP-CCMV} + gel_{GFP-ELP-CCMV} / mw_{GFP-ELP-CCMV}} \quad \text{Eq. (S1)}$$

where gel is the intensity of the protein band on the SDS-PAGE gel as determined by ImageJ analysis; mw is the molecular weight of the protein.

2 Experimental supplemental discussions

2.1 Optimization of conditions to allow for optimal dynamics

In order to study VW1-VW8 ELP-CCMV capsid size shifts, we first investigated the optimal conditions that would allow for the dynamic behavior of the capsids. In preliminary results we observed a size shift from *T* = 1 capsids to *T* = 3 capsids during overnight dialysis from pH 7.5 to pH 5.0 at 4 °C (Figure S1). We, therefore, evaluated with SEC whether the reverse size shift would also take place. We observed that only a partial shift from *T* = 3 to *T* = 1 capsids took place during the overnight dialysis to pH 7.5 buffer with 100 mM NaCl (Figure S1A,B first two chromatograms in each panel). We then proceeded by investigating whether a second overnight dialysis to pH 5.0 would induce a re-shift back to *T* = 3 capsids as well. Interestingly, this was the case only when the dialysis was performed at 4 °C (Figure S1B), while at 21 °C no size shift was observed. This indicates that at 21 °C the capsids are much less dynamic than at 4 °C. Although this seems contra-intuitive at first, this observation can be explained by the interactions between the hydrophobic ELP-domains which are much stronger at 21 °C than at 4 °C. So it is highly likely that these interactions in the capsid interior hamper rearrangements of the CP domains in the capsid shell, which are necessary for a size shift.

Another factor that was thought to influence the capsid dynamics as a result of ELP interactions is the ionic strength of the buffers used. Previously, we used a pH 7.5 buffer with 100 mM NaCl to completely disassemble other ELP-CCMV variants. Therefore, we hypothesized that this ionic strength would also allow for dynamics within capsids of our more hydrophobic VW1-VW8 ELP-

CCMV variant. As, ideally, we wanted to study the capsid size shifts while varying as few factors as possible, preferably only the pH, we attempted to store $T = 3$ capsids in pH 5.0 buffer with 100 mM NaCl instead of 500 mM NaCl. However, this, unfortunately, led to the aggregation of the

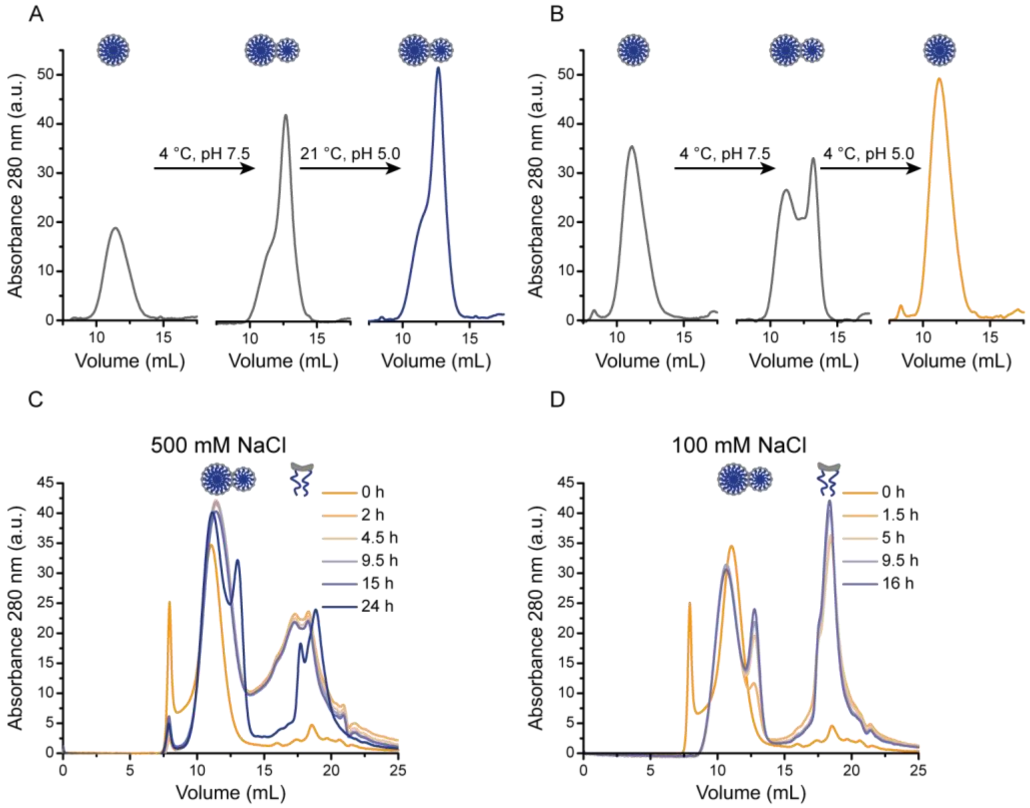

Figure S1. Optimization of dynamics during VW1-VW8 ELP-CCMV capsid size changes. A and B) Optimization of temperature. A $T = 3$ capsid solution was dialyzed overnight at 4 °C to pH 7.5 buffer with 100 mM NaCl, yielding a mixture of $T = 3$ and $T = 1$ capsids. Subsequently, the capsid mixture was dialyzed back to pH 5.0 at 21 °C (A) or 4 °C (B), only resulting in a complete shift back to $T = 3$ capsids at the lower temperature. The capsid size was monitored after each dialysis step with SEC via the protein absorbance at 280 nm. C and D) Optimization of NaCl concentration. A $T = 3$ capsid solution was dialyzed overnight at 4 °C to pH 7.5 buffer with 500 mM (C) or 100 mM (D) NaCl. The capsid size was monitored during dialysis with SEC via the protein absorbance at 280 nm. Aggregated proteins or higher order assemblies elute around 7 mL, $T = 3$ capsids around 11 mL, $T = 1$ capsids around 13 mL, and coat protein dimers around 17 mL.

protein already within 16 hours (data not shown). We, therefore, evaluated whether VW1-VW8 ELP-CCMV exhibited dynamic behavior when dialyzed from pH 5.0 buffer with 500 mM NaCl to pH 7.5 buffer with 500 mM NaCl, thus only changing the pH. Unfortunately, only a very small part of the capsids appeared to be shifted in size after 24 hours (Figure S1C) as compared to dialysis to pH 7.5 buffer with 100 mM NaCl (Figure S1D), which can again be explained by hydrophobic interactions between ELP domains hampering capsid dynamics at 500 mM NaCl. We, therefore,

decided to use a shift from pH 5.0 with 500 mM NaCl to pH 7.5 with 100 mM NaCl and vice versa to study the dynamics during VW1-VW8 ELP-CCMV capsid size decrease and increase respectively as is discussed further in the main text.

2.2 Optimization of dialysis conditions

As described in the previous section, it is necessary to both change the pH and NaCl concentration in order to study size shifts of VW1-VW8 ELP-CCMV capsids. If only the pH would have to be adjusted, this could have been done by adding either HCl or NaOH to the buffer. However, to also adjust the NaCl concentration a buffer exchange step is necessary. Although spin-filtration would be the quickest option and would allow for evaluation of the capsid size upon change of the conditions very quickly, it would also introduce changes in the protein concentration, which could affect the capsid assembly state. As this could complicate our evaluation of capsid size changes as a function of pH, we decided to perform dialysis in order to change the pH and NaCl concentration, despite being a slower process than spin-filtration.

During initial experiments, it was observed that there is a large dependency of capsid dynamics on dialysis time while incubation periods at 4 °C were kept constant (Figure S2A). When a *T* = 3

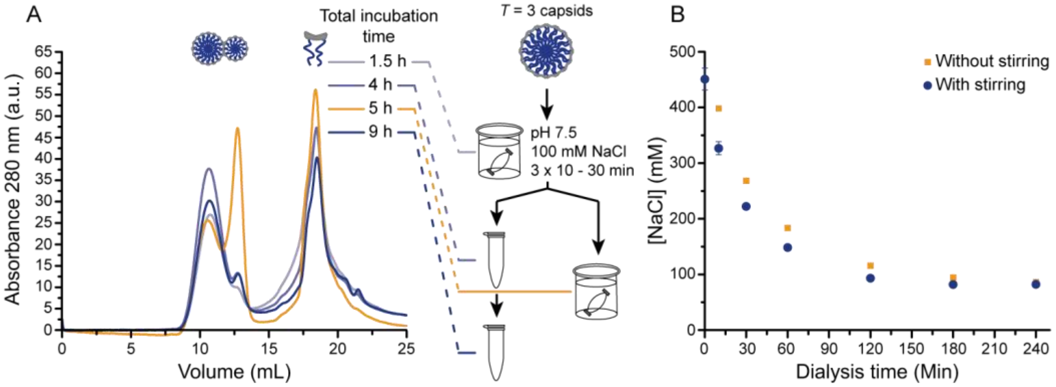

Figure S2. Optimization of dialysis conditions. A) SEC chromatograms of samples taken during prolonged dialysis at 4 °C in pH 7.5 buffer with 100 mM NaCl (5 h, orange line) or during incubation in an Eppendorf tube at 4 °C in pH 7.5 buffer with 100 mM NaCl after 3 x 10 minutes initial dialysis (1.5 h, 4 h, 9 h). Thus, while the total incubation time at 4 °C in pH 7.5 buffer with 100 mM NaCl was similar for the 4 h and 5 h samples, the dialysis time was much shorter for the 4 h sample. B) Reduction of the NaCl concentration during dialysis of pH 7.5 buffer with 500 mM NaCl to pH 7.5 buffer with 100 mM NaCl at 4 °C. Buffer was exchanged after 30 and 60 minutes and the dialysis buffer was either stirred at 150 rpm (blue circles) or not stirred (yellow squares). All measurements were performed in triplicate and data is presented as mean ± standard deviation.

capsid solution was dialyzed to pH 7.5 buffer with 100 mM NaCl, a large shift to *T* = 1 capsids was only observed during SEC analysis when the total dialysis time was more than 30 minutes. This indicated that either the pH or the NaCl concentration changed slower than anticipated during

dialysis. As the pH switch during dialysis takes place within minutes, it was suspected that the other variable during dialysis, the NaCl concentration, changed more slowly. The amount of NaCl that is dissolved in an aqueous solution affects the conductivity of that solution, thus conductivity measurements were performed to follow the change of the NaCl concentration during dialysis. Hereto, a mock dialysis with the same ratio between the volume inside the dialysis bag and the solvent volume was performed and the conductivity was monitored over a time course of 4 hours (Figure S2B). A dialysis time of around 2 hours was necessary to fully convert the NaCl concentration from 500 mM to 100 mM NaCl in the dialysis bag, which explains why dialysis time is such an important determinant of capsid dynamics.

2.3 Optimization of the SEC protocol for studying the self-assembly dynamics during size reduction

As during initial dynamics experiments large quantities of dimers were observed in the SEC chromatograms when pH 7.5 buffer with 100 mM NaCl was the eluent, while these were never observed for VW1-VW8 ELP-CCMV before, the origin of these dimers was evaluated. Hereto, a 100 µM VW1-VW8 ELP-CCMV coat protein solution in pH 5.0 buffer was dialyzed (MWCO 12-14 kDa) to pH 7.5 buffer with 100 mM NaCl at 4 °C overnight and subsequently spiked with known amounts of native ELP-CCMV dimers in the same buffer. DLS and native PAGE were employed to analyze the capsid-dimer mixtures. From the DLS results in Figure S3A and B, it becomes clear that DLS is not sensitive enough to detect dimers in capsid-dimer mixtures, which could be explained by the high scattering of the capsids overpowering any light scattering caused by the much smaller dimers. Therefore, although no dimers are detected with DLS of the dialyzed capsids, this does not confirm that indeed no dimers are present in this capsid solution. We therefore focused on native PAGE analysis. From the results in Figure S3C, it can be appreciated that capsids and dimers can be easily distinguished from each other. Furthermore, based on the band intensities on the gel it can be stated that in the VW1-VW8 ELP-CCMV capsid solution less than 5 % dimers are present. This indicates that the large fractions of dimers that are observed in the SEC chromatogram are most likely the result of some disassembly of VW1-VW8 ELP-CCMV capsids taking place during SEC analysis, which might be caused by the extreme dilution (240 times) during the chromatographic procedure.

As for data analysis purposes, the SEC chromatograms should provide the best representation of the assembly state of VW1-VW8 ELP-CCMV during dynamics, a protocol was developed for

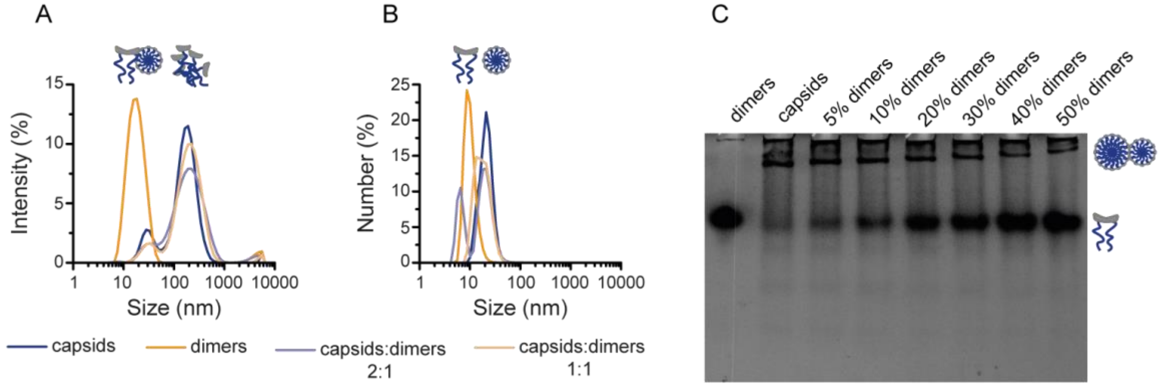

Figure S3. Analysis of dimers in capsid mixtures. A and B) DLS analysis of capsids, dimers and capsid-dimer mixtures. Both the intensity (A) and number (B) distributions do not allow for reliable estimation of the number of dimers in the capsid-dimer mixtures. C) Native PAGE analysis of capsids, dimers and capsid-dimer mixtures.

inhibiting capsid dynamics prior to SEC analysis. Hereto, a 100 µM VW1-VW8 ELP-CCMV solution in pH 5.0 buffer was dialyzed (MWCO 12-14 kDa) to pH 7.5 buffer with 100 mM NaCl at 4 °C overnight and subsequently incubated with various amounts of $NiCl_2$ for 50 minutes (the duration of one SEC run) at room temperature.[6] From the results in Figure S4A and B, it can be observed that the addition of at least 0.2 equivalents Ni^{2+} (relative to the amount of VW1-VW8 ELP-CCMV coat protein concentration) successfully reduces the number of dimers that are observed in the SEC chromatograms, without affecting the $T = 3 : T = 1$ ratios. With increasing amounts of Ni^{2+} also some higher-order structures became visible on the SEC chromatograms (around 7 mL), indicating that high amounts of Ni^{2+} alter the protein fractions in the VW1-VW8 ELP-CCMV solution. Therefore, the addition of 0.2 equivalents of Ni^{2+} was found to be most suitable to reduce the number of dimers introduced due to dilution on the SEC column while not affecting the protein fractions. To confirm that the addition of this amount of Ni^{2+} effectively stops any dynamics, a sample that was dialyzed for 30 minutes was subjected to SEC analysis after 50 minutes or 7 hours of incubation with Ni^{2+} (Figure S4C,D). The resulting SEC traces and protein fractions were very similar, indicating that the addition of 0.2 equivalents of Ni^{2+} effectively stops the dynamics and stabilizes the samples for prolonged storage prior to SEC analysis.

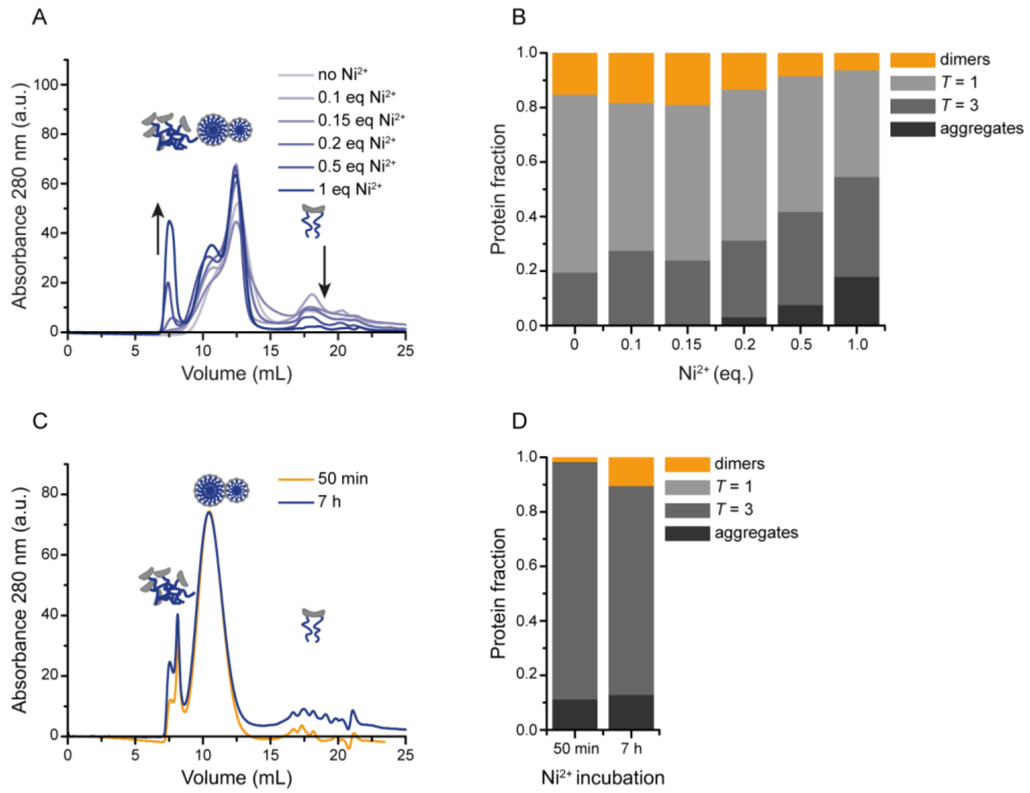

Figure S4. Optimization of SEC conditions. A) SEC analysis of capsid mixtures that were stabilized with various amounts of Ni2+. Samples were incubated with the indicated amount of Ni2+ for 50 minutes at room temperature prior to injection. B) Quantification of the protein fraction as dimers, T = 1 capsids, T = 3 capsids and aggregates based on the SEC chromatograms in figure A. C) SEC analysis of capsid mixtures that were stabilized with 0.2 equivalents of Ni2+ for 50 minutes or 7 hours at room temperature prior to injection. D) Quantification of the protein fraction as dimers, T = 1 capsids, T = 3 capsids and aggregates based on the SEC chromatograms in figure C.

3 Theoretical methods and discussions

3.1 Theory

Based on previous experimental, theoretical and computer simulation results, we put forward that nucleation is the underlying mechanism for capsid assembly and disassembly.[7–9] To this end, we combine equilibrium theory, borrowed from the physics of supramolecular polymers, and classical nucleation theory.[10] This allows us to calculate the time evolution of the assembly and disassembly of mixtures of capsids, the predictions of which we compare with our experimental results. Note that the assembly kinetics of free subunits into competing capsids with different T numbers has been discussed before [11,12] - a kinetic theory of T number conversion has not yet been attempted.

To obtain the thermodynamic parameters required for our kinetic theory, we write the free energy of an aqueous solution in which only free ELP-CCMV subunits and the fully formed capsids are allowed to be present. In the equilibrium theory we ignore the intermediate states, as previous experimental and simulation work, as well as the findings presented in this paper clearly show that they are barely detectable (if at all) and represent short-lived states.[13–17] Within a mean-field approximation, the dimension-less free energy f per solvent molecule of a dilute, aqueous solution, in which the mole fraction of ELP-CP subunits is x_s and that of the fully formed capsids x_1 for the $T = 1$ and x_3 for $T = 3$ species can be written as

$$f = x_s \, ln \, x_s - x_s + \sum_{T=1,3}[x_T \, ln \, x_T - x_T + q_T g_T x_T] \qquad \text{Eq. (S2)}$$

in units of the thermal energy $k_B T$ with k_B Boltzmann's constant and T the absolute temperature. See Refs.[10,18] for the derivation of Eq. (S2). The effective binding free energy between ELP-CP subunits in $T = 1$ and $T = 3$ capsids are represented by g_1 and g_3 in units of thermal energy, averaged over all subunits of a fully formed capsid. Finally, q_1 and q_3 represent the number of subunits that make up the capsid of the $T = 1{,}3$ shells, respectively. The first four terms in Eq. (S2) represent the translational entropy and the entropy of mixing while the last term accounts for the overall (net) binding free energy of the subunits in assemblies.

An important ingredient in the development of the classical nucleation theory for virus capsids, is the difference between the chemical potential of free protein subunits in the metastable solution and bound proteins in the capsids. The chemical potential of the free protein subunits in solution, $\mu_s = \partial f/\partial x_s$, and chemical potentials of the capsids, $\mu_T = \partial f/\partial x_T$, follow from Eq. (S2) where $T = 1{,}3$ indicate the T number of the shell. The equilibrium distribution of proteins over the free proteins and different types of capsids can be obtained by minimizing Eq. (S2) subject to the condition of conservation of mass, $c_s = x_s + q_1 x_1 + q_3 x_3$, with c_s the overall mole fraction of coat proteins in solution. The resulting mass-action equations are $x_T = (x_s e^{-g_T})^{q_T}$ with $T = 1{,}3$. The reference chemical potentials are tacitly absorbed in the binding free energies g_T. Since x_1 and x_3 are always (much) smaller than unity, both $x_s e^{-g_1}$ and $x_s e^{-g_3}$ should also be smaller than unity, *i.e.*, x_s can never exceed $c^*{}_1 = e^{g_1}$ or $c^*{}_3 = e^{g_3}$. Thus, for each type of capsid, there is a critical free protein concentration $c_T{}^*$ below which the concentration of capsids is almost zero as the number of subunits in the capsids, q_T, is large compared to unity. For CCMV, the basic protein subunits are dimers, so $q_1 = 30$ for the $T = 1$ and $q_3 = 90$ for the $T = 3$ capsid.

Using the equilibrium theory described above, we can now set up the kinetic theory of capsid assembly and disassembly within the framework of CNT.[8] The Gibbs free energy of the formation of an incomplete spherical capsid of the T species containing $n_T = 1,...,q_T$ molecules with a circular rim can be written as

$$\Delta G_T(n_T) = n_T\, \Delta\mu_T + a_T\sqrt{q_T(q_T - n_T)}, \qquad \text{Eq. (S3)}$$

where $a_T = 4\pi R_T \sigma_T / q_T$ is a dimensionless magnitude of the rim energy, with R_T the radius of the shell and σ_T the free energy cost per unit length of the rim.[10] σ_T can be estimated as $\sigma_T = \frac{-s g_T}{r_T}$ where $s \in [0,1]$ is a geometric factor indicating the average fraction of bonds that a subunit on the rim is missing, which depends on the local coordination number and roughness of the rim. r_T is the effective diameter of a protein unit that is approximated as a disk. Assuming that the surface of a fully formed capsid is covered entirely by capsid proteins, the effective diameter can be written as $r_T = \frac{\sqrt{q_T}}{4R_T}$.[8] A previous and more detailed study on the line tension of shells composed of Lennard-Jones disks, packed on the surface of sphere, shows that $s \approx 0.3 - 0.4$, with the latter value an upper limit as proteins are more flexible than Jennard-Jones particles - we set $s = 0.3$ in our simulations.[19] Finally, the first term in Eq. (S3) represents the thermodynamic driving force for the assembly or disassembly of capsids. To obtain the (dimensionless) barrier height, $\Delta G_T{}^*$ for the two T numbers, we calculate the critical nucleus size $n_T{}^*$, that is, find the value of n_T for which Eq. (S3) is maximal and insert this into Eq. (S3) to obtain

$$\Delta G_{as,T}{}^* = \Delta G_T{}^0\left(\sqrt{\Gamma_T{}^2 + 1} - \Gamma_T\right), \qquad \text{Eq. (S4)}$$

where $\Delta G_T{}^0 \equiv q_T a_T / 2$ is the maximum barrier height and $\Gamma_T \equiv -\Delta\mu_T / a_T$ is a measure for the degree of super- or undersaturation for the species T. We note that Γ_T depends on the concentration of a capsid.

In the process of assembly of a capsid, subunits can attach to and detach from the growing shell through a sequence of what we presume to be reversible kinetic steps. Within the classical nucleation theory, the rate of capsid assembly is limited by the rate of the formation of the energetically most unfavorable critical nucleus through the Boltzmann weight $\Delta G_T{}^*$ that acts as a kinetic bottleneck.

The steady-state nucleation rate for *association* reads[10]

$$J_{as,T} = x_s\, v^*{}_T Z_T\, exp\,(-\Delta G^*{}_{as,T}), \qquad \text{Eq. (S5)}$$

where $Z_T = \sqrt{-\frac{1}{2\pi}(\partial^2 \Delta G_T/\partial n^2)_{n=n_T^*}} = \sqrt{\frac{a_T}{q_T \pi}}(1+{\Gamma_T}^2)^{3/4}$ is the so-called Zeldovich factor that describes the sharpness of the free energy barrier and that may be interpreted as a measure of the lifetime of the critical nucleus of size ${n_T}^*$.[13] The attempt or attachment frequency ${\nu^*}_T$ of the monomers attaching to the critical nucleus depends on the mode of attachment, and may be , *e.g.*, a function of the diffusivity and concentration of the free monomers, the size of critical nucleus, and on some internal molecular time scale associated with the docking process that may depend on conformational switching.[20] For simplicity, we assume that it does not depend on the size of the clusters nor on the concentration.

To model the disassembly process, we presume that the initial state constitutes a fully formed capsid. Within classical nucleation theory, the rate of disassembly of a capsid is limited by dissociation of the critical nucleus, *i.e.*, it depends on the time required to jump over the height of the free energy barrier ${\Delta G_{dis,T}}^*$ albeit in the opposite direction from the assembly process. The nucleation rate for disassembly of complete capsids of species T reads

$$J_{dis,T} = x_T \, {\nu^*}_T Z_T \, exp\,(-\Delta {G^*}_{dis,T}) \, , \qquad \text{Eq. (S6)}$$

where ${\Delta G_{dis,T}}^*$ represents the free energy barrier for the disassembly of a shell to form monomers. Notice that the dissociation rate depends on x_T, the capsid concentrations of species $T = 1{,}3$. We shall presume that the attachment frequency associated with the association process is the same as that of the dissociation process, as it describes the same process and we presume it to be independent of the size of the critical nucleus.[21]

Because capsids with different T numbers have different radius of curvature, we do not allow for a direct transition from one T number to another one. In our allowed reaction path pathway, growth or disassembly can only proceed by the shedding or docking of individual protein subunits, which for CCMV constitute coat protein dimers. This is not a far-fetched reaction path, as our experiments show no indication of partially disassembled $T = 3$ particles spontaneously morphing into $T = 1$ particles, or vice versa, $T = 1$ particles opening up to absorb subunits and growing into a $T = 3$ particles. Hence, we presume that, first, one type of capsid disassembles into dimers, second, free dimers reassemble into different capsid sizes following their corresponding assembly nucleation rates.

Presuming that kinetic processes are sufficiently slow to allow us to use the expressions for steady-state nucleation rates for association and dissociation, *i.e.*, presume a quasi steady state

to hold, the set of equations describing the concentration of dimers, $T = 1$ and $T = 3$ capsids in our system can finally be expressed as follows,

$$\frac{dx_s}{dt} = -q_1 J_{as,1} - q_3 J_{as,3} + q_1 J_{dis,1} + q_3 J_{dis,3} \ , \qquad \text{Eq. (S7)}$$

and

$$\frac{dx_T}{dt} = J_{as,T} - J_{dis,T}. \qquad \text{Eq. (S8)}$$

The quantities on the left-hand sides of Eqs. (S7 and S8) represent time derivatives of the concentrations of the three species in our model. The terms on the right-hand sides are due to the changes in the formation or dissociation of capsids, where we let all concentrations be time dependent yet obey mass conservation at all times. We solve the above system of equations numerically, using an explicit forward Euler method with adaptive time steps. (See SI 3.2 for more information.)

From the solutions of these equations, we calculate the fraction of dimers in each type of capsid compared to the total number of dimers *in all capsids*,

$$f_T = \frac{q_T x_T}{\sum_{i=1,3} q_i x_i} \qquad \text{Eq. (S9)}$$

where $T = 1{,}3$ depending on the T number. In general, the time steps in the simulations depend on various parameters, such as the attempt frequency, binding energies and size of the capsids. In order to fit the theory to the experimental data, we choose one experimental data point in the time series of the disassembly of $T = 3$ and the assembly of $T = 1$, and use it as our reference point. When we find the same ratio of proteins in the two kinds of capsid in our simulations as that in the experiments, we set the time in the simulations equal to the time in the experiments. Next, we rescale all other simulation data points accordingly. We repeat this process for the disassembly of $T = 1$ and the assembly of $T = 3$. To check the robustness of this technique, we consider different data points as the reference point. The agreement between theory and experiments does not depend strongly on the reference point that we take. (See the SI 3.4 for more information.)

It is important to realize that the time that it takes to change the pH and the salt concentration of the buffer solution might not be exactly the same in each experiment. In addition, the lag time for assembly and disassembly of capsids with different sizes are arguably different. Therefore it is difficult to pinpoint the actual “time zero” for each individual experiment. In order to deal with this uncertainty, we start collecting data 30 minutes after the experiment commences. We also assume that the lag times are negligible on the time scale of the experiment, thus we ignore the

first phase of assembly in CNT in which capsids have not started to assemble or disassemble.[10]

3.2 Numerics

The kinetics equations predicted by CNT (Eqs. S7 and S8) are solved by using finite difference methods. Assembly and disassembly nucleation rates at the beginning of the simulation are determined by the initial conditions. The concentrations of capsids and free dimers are calculated at each time step, using the values and nucleation rates at the previous time step. Hence, our time-stepping equations read:

$$x_s{}^{t+\Delta t} = x_s{}^t + (-q_1 J^t{}_{as,1} - q_3 J^t{}_{as,3} + q_1 J^t{}_{dis,1} + q_3 J^t{}_{dis,3}) \times \Delta t \,, \qquad \text{Eq. (S10)}$$

and

$$x_T{}^{t+\Delta t} = x_T{}^t + (J^t{}_{as,T} - J^t{}_{dis,T}) \times \Delta t, \qquad \text{Eq. (S11)}$$

where $x_s{}^t$ and $x_T{}^t$ are the concentrations of free dimers and capsids of size T at time t; q_1 and q_3 represent the number of subunits in each fully formed capsid of size $T = 1$ and $T = 3$. As already alluded to, we assume that dimers are our building blocks, so that $q_1 = 30$ and $q_3 = 90$. The instantaneous steady-state assembly and disassembly nucleation rates of capsid size T at time t are $J^t{}_{as,T}$ and $J^t{}_{dis,T}$, respectively.

The nucleation rates depend highly on the free dimer concentration. In order to speed up the simulation, we use an adaptive time step such that a maximum of 0.01% of free dimers at time t can be consumed by growing shells. Similarly, a maximum of 0.01% of free dimers can be released by dissociated shells at time t. In other words,

$$\Delta t = \frac{0.0001\ x_s}{Max(J^t{}_1, J^t{}_3)} \qquad \text{Eq. (S12)}$$

where $J^t{}_T = \left|J^t{}_{as,T} - J^t{}_{dis,T}\right|$ is the absolute assembly or disassembly rate of capsid size T. The simulation continues until full depletion of the unfavorable capsid size.

From equilibrium theory and experimental observations, we have to assume that there are some free dimers remaining in the solution before the quench, that is, before the induced shift in pH and in salt concentration that on the time scale of the experiment is (virtually) instantaneous. Therefore, we invoke a non-zero value as our initial free dimer concentration. Quickly after starting

the simulation, the dimer concentration converges to a fixed concentration relatively close to what must be the smaller critical concentration. Having initially more dimers in the system leads to the fast formation of capsids. On the other hand, a low dimer concentration at the start of the simulation increases the initial disassembly rate. In order to avoid both of these conditions, we choose the initial dimer concentration near the concentration it converges to. It also helps us to avoid any divergence in the simulation as the dissociation rates increase significantly at low dimer concentrations.

Based on the dimer concentration at the end of the experiment, which is relatively close to the critical concentration of the more stable species, we approximate the total dimer concentration is around 10 times larger than the critical concentration. Therefore, the overall protein in the unfavorable capsid we set at $10\ c^*$, where $c^* = Min(c^*_1, c^*_3)$. (See table S4 and S5 for parameter values.)

Due to the universality of the phenomena, the model is capable of reproducing the experimental results by using different binding energies. We decide to fix the binding energy of T=1 in our framework and generate experimental results only by changing the binding energy of T=3. This allows us to have a better comparison between the two types of experiment.

3.3 The fraction of protein dimers in free solution and in capsids

In the main text we show results of the fraction of proteins in the two types of capsid, f_T, as we

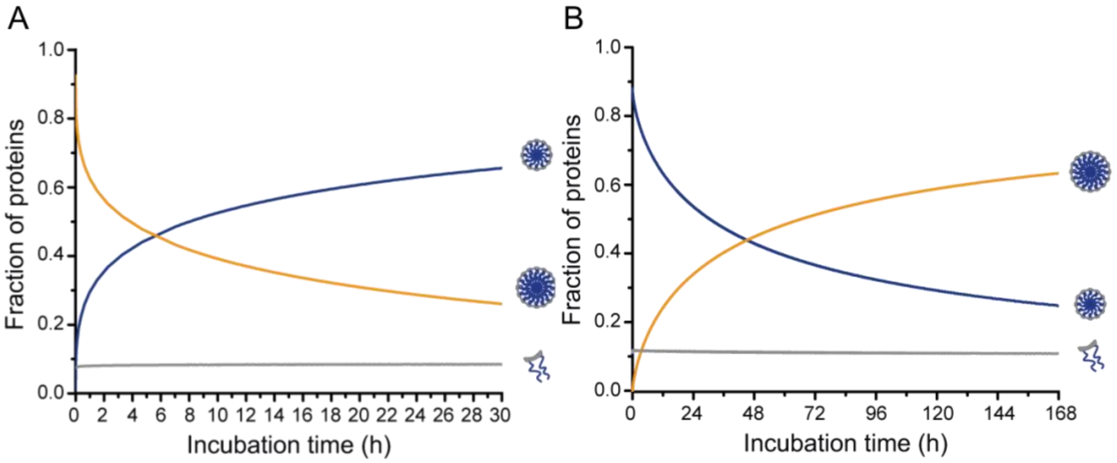

Figure S5. The fraction of protein in T=1 (blue line) and T=3 (yellow line) and dimer (gray line) over the total amount of protein in the solution (h_T) for the experiments showing A) disassembly of T=1 (represented in the figure 3) and B) disassembly of T=3 (represented in the figure 2). Refer to table S4 and S5 for the parameter values.

find this the most informative. Alternatively, we can also calculate the fraction of dimers in one type of capsid relative to the *total* number of dimers in solution,

$$h_T = \frac{q_T x_T}{c_s}, \qquad \text{Eq. (S13)}$$

where $T = 1,3$ is as before the T number of the capsid and c_s the overall dimer concentration in solution. Our simulations show that for our choice of parameters the concentration of dimers remains constant during the process (see Figure S5), implying that the increase in the protein fraction in capsids is not due to the assembly of the free dimers initially present in the solution. This agrees with what is seen in the experiments, see Figure S7. This supports our suggestion that one capsid size disassembles into free dimers and that these proteins re-assemble into the other capsid size, and that the fraction f_T is the relevant quantity describing the assembly and disassembly kinetics for the problem in hand.

3.4 Fitting of the simulation results with respect to different reference points

We have calibrated the simulation results using a reference point in the data series, as mentioned in the caption for figure 2C and 3C. Here we re-calibrated the same simulation data set with respect to a number of reference points to verify the robustness of our fitting procedure. We find that the curve fits depend only relatively weakly on the choice of reference point (Figure S6). Unfortunately, our numerical implementation of Classical Nucleation Theory does not allow us to find the fundamental time scale, that is, the time scale associated with the attempt frequency. In spite of this, we are able to show that the disassembly and assembly of the two different capsid sizes can be explained by CNT.

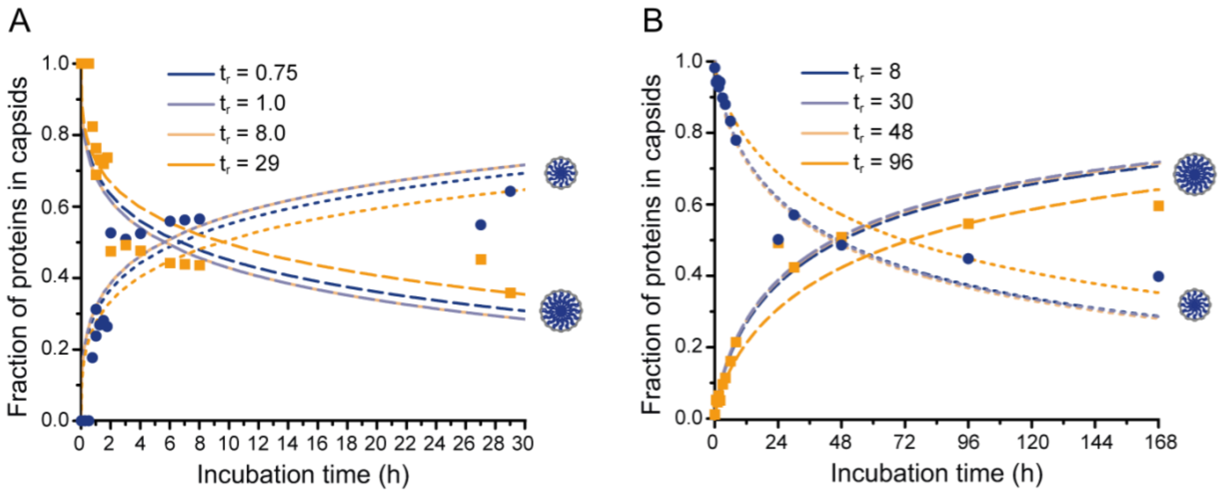

Figure S6. The fraction of protein in capsids (f_T as described in Eq S8) of T=1 (short dashed lines) and T=3 (long dashed lines) for A) disassembly of T=3 (represented in the figure 2) and B) disassembly of T=1 (represented in the figure 3) experiments, using different reference times. See table S4 and S5 for parameter values.

3.5 Table of parameters

All parameters related to the simulation of disassembly of T=3 and assembly of T=1, and vice versa, discussed in the main text and used in our comparison with the experiments are tabulated below.

Table S4 – Parameters used in simulation of disassembly of T=3 and assembly of T=1

Parameter	Value (unit)	Description
g_1	-15 (k_BT)	Binding energy of T=1 capsids
g_3	-14.7 (k_BT)	Binding energy of T=3 capsids
q_1	30	Number of dimers in fully formed T=1
q_3	90	Number of dimers in fully formed T=3
x_s(t=0)	0.8 c^*_1	Initial dimer concentration
x_1(t=0)	0	Initial T=1 concentration
x_3(t=0)	$\frac{1}{9}c_1^*$	Initial T=3 concentration
ν^*	1 (a.u.)	Critical attempt frequency
s	0.3	fraction of bonds that a rim protein has fewer than a core protein
t_r	4 (hours)	Reference time

Table S5 – Parameters used in simulation of disassembly of T=1 and assembly of T=3

Parameter	Value (unit)	Description
g_1	-15 (k_BT)	Binding energy of T=1 capsids
g_3	-15.4 (k_BT)	Binding energy of T=3 capsids
q_1	30	Number of dimers in fully formed T=1
q_3	90	Number of dimers in fully formed T=3
$x_s(t=0)$	1.3 c^*_3	Initial dimer concentration
$x_1(t=0)$	$\frac{1}{3}c_3^*$	Initial T=1 concentration
$x_3(t=0)$	0	Initial T=3 concentration
ν^*	1 (a.u.)	Critical attempt frequency
s	0.3	fraction of bonds that a rim protein has fewer than a core protein
t_r	48 (hours)	Reference time

4 Supplemental figures

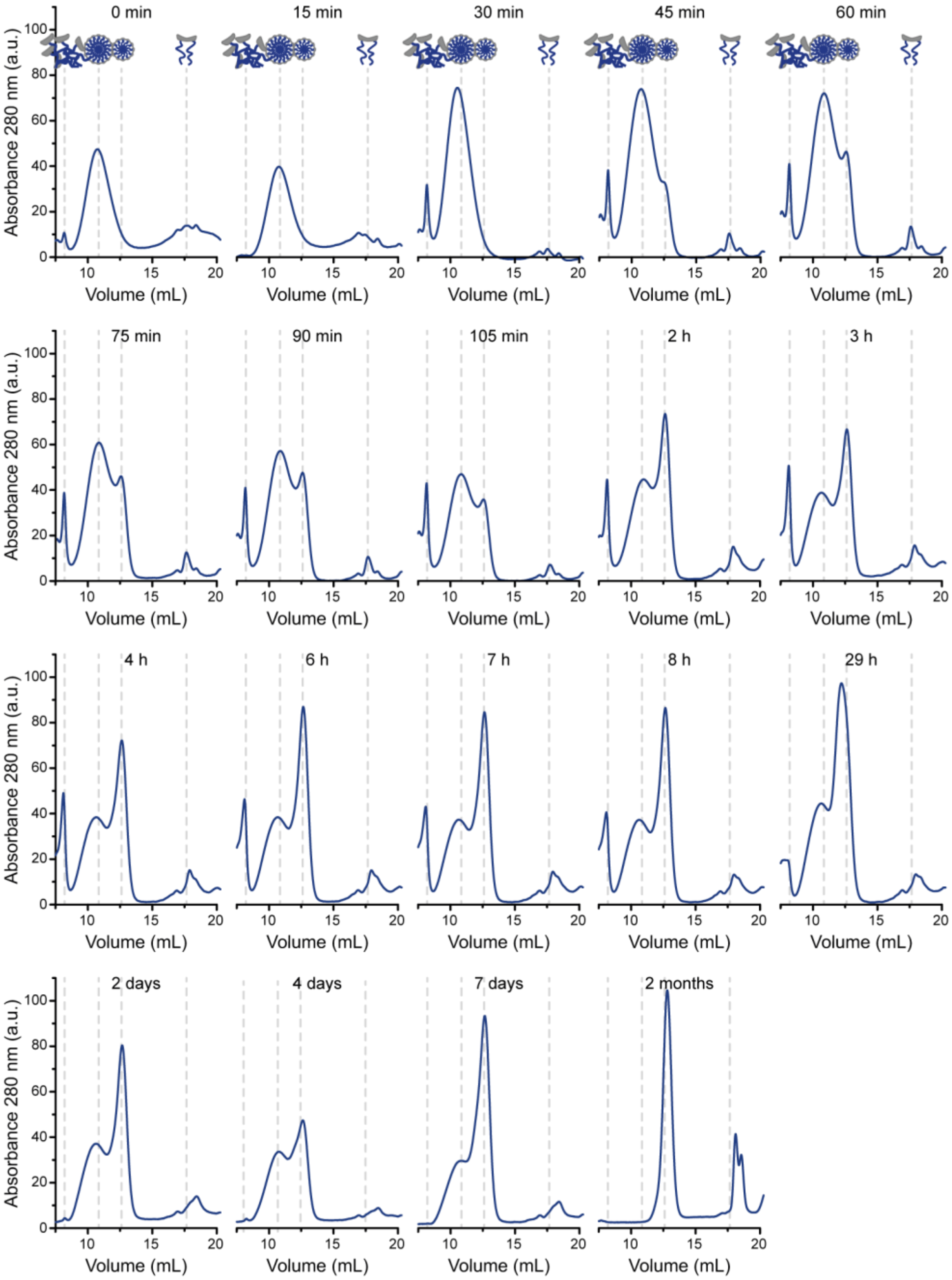

Figure S7. SEC analysis of the shift from $T = 3$ to $T = 1$ capsids (dataset 1). SEC chromatograms were measured after the indicated dialysis times to pH 7.5 buffer with 100 mM NaCl at 4 °C. Samples were stabilized by incubation with 0.2 eq. of Ni^{2+} for 50 minutes at room temperature prior to analysis. The results from three separate experiments are combined: 0 to 105 minutes, 2 h to 29 h and 2 days to 2 months.

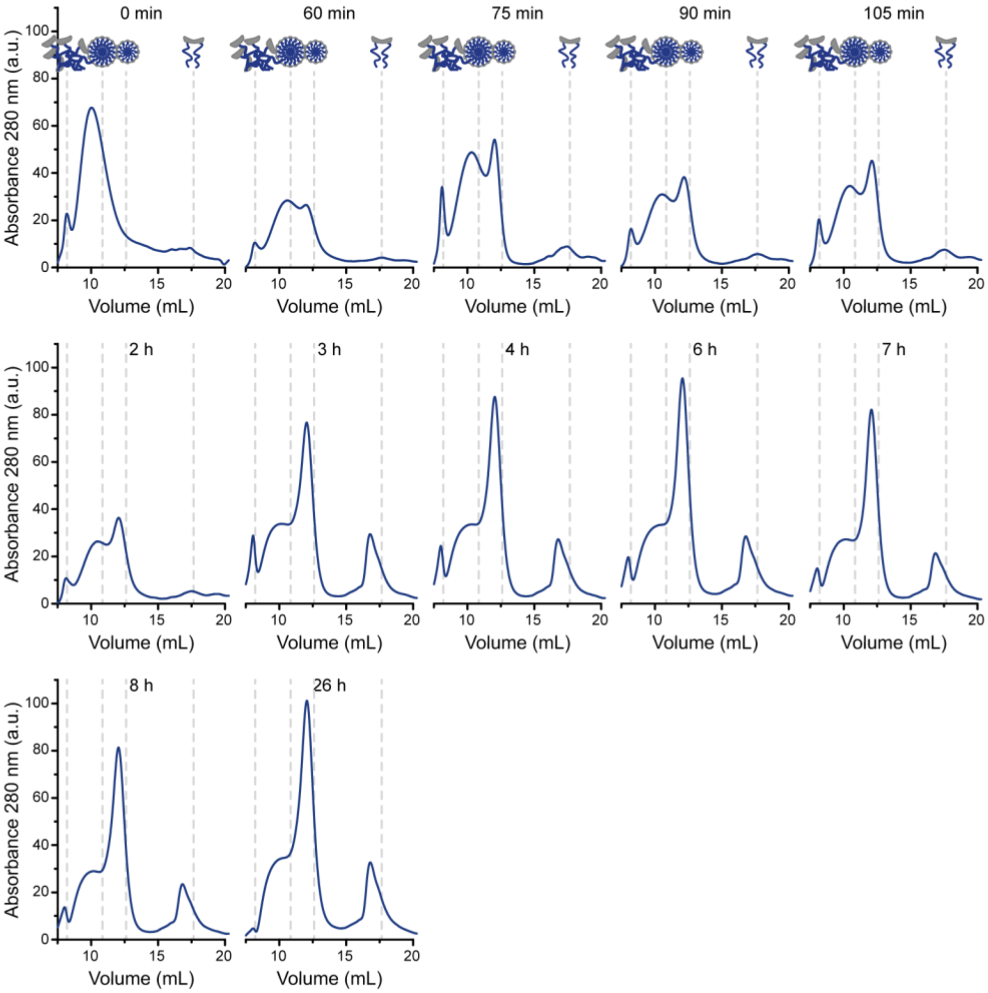

Figure S8. SEC analysis of the shift from T = 3 to T = 1 capsids (dataset 2). SEC chromatograms were measured after the indicated dialysis times to pH 7.5 buffer with 100 mM NaCl at 4 °C. Samples were stabilized by incubation with 0.2 eq. of Ni^{2+} for 50 minutes at room temperature prior to analysis. The results from two separate experiments are combined: 0 to 2 h and 3 h to 26 h.

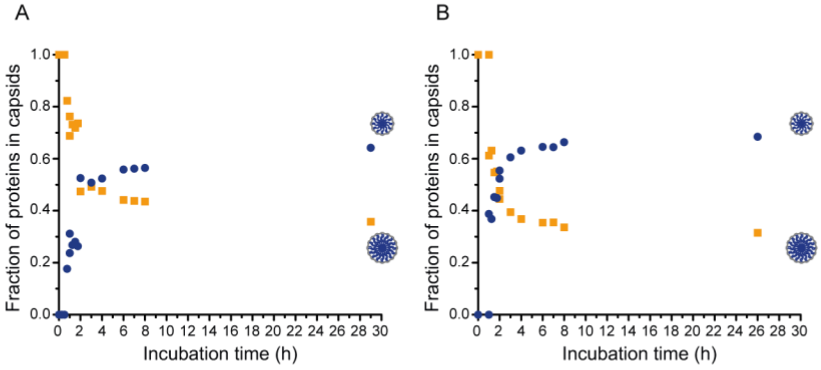

Figure S9. Comparison of time curves based on both datasets for the shift from T = 3 to T = 1 capsids. Protein fractions as T = 1 (blue circles) and T = 3 (yellow squares) capsids as determined by integration of the SEC chromatograms of Figure S7 (A) and Figure S8 (B).

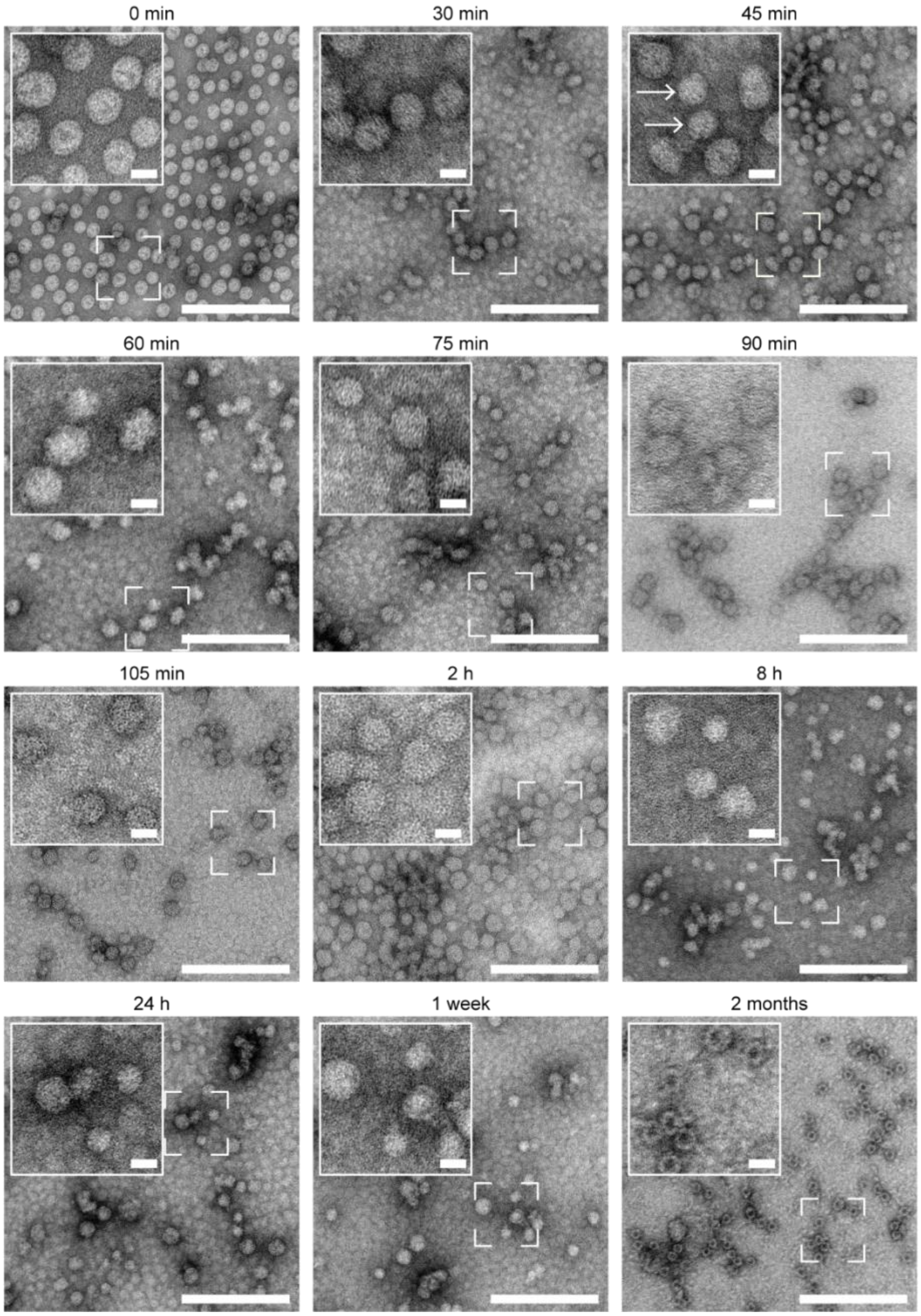

Figure S10. TEM analysis of VW1-VW8 ELP-CCMV capsids during size decrease in pH 7.5 buffer with 100 mM NaCl. Samples were taken after the indicated dialysis times at 4 °C and negatively stained with uranyl acetate. Areas indicated with brackets are displayed as zoomed images with $T = 1$ capsids indicated by arrows. Scale bars correspond to 200 nm (main images) and 20 nm (zoomed images).

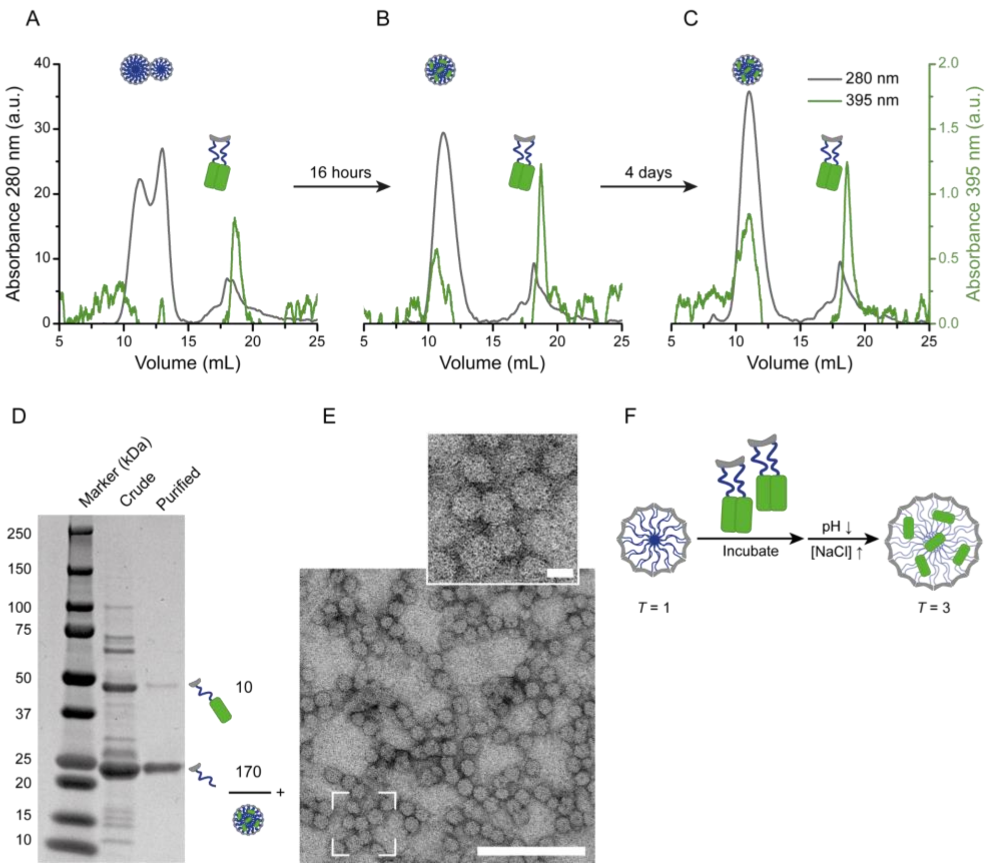

Figure S11. Analysis of mEGFP-labeling of *T*=3 VW1-VW8 ELP-CCMV capsids by mixing empty *T*=1 capsids with labeled mEGFP-VW1-VW8 ELP-CCMV dimers. A-C) SEC chromatograms of 25 μM mEGFP-VW1-VW8 ELP-CCMV and 100 μM VW1-VW8 ELP-CCMV capsid mixtures in pH 5.0 buffer with protein absorbance measured at 280 nm (gray curve and axis) and mEGFP absorbance measured at 395 nm (green curve and axis). Mixtures were allowed to mix at 4 °C, pH 5.0 for 90 minutes (A), overnight (B) or 4 days (C). The increase in mEGFP signal indicates that the mEGFP-labeled dimers exchange with non-labeled dimers over time. D) SDS-PAGE analysis of SEC purification after 4 days of exchange. The gel was stained with Coomassie Brilliant Blue. ImageJ quantification is depicted next to the gel. E) Uranyl acetate-stained TEM micrograph of SEC-purified capsids. The area indicated with brackets is displayed as a zoomed image. Scale bars correspond to 200 nm (main image) and 20 nm (zoomed image). F) Schematic of the process we studied: empty *T*=1 capsids are mixed with mEGFP-VW1-VW8 ELP-CCMV dimers, which upon a decrease in pH and increase in NaCl concentration exchange with non-labelled dimers to form labeled *T*=3 capsids.

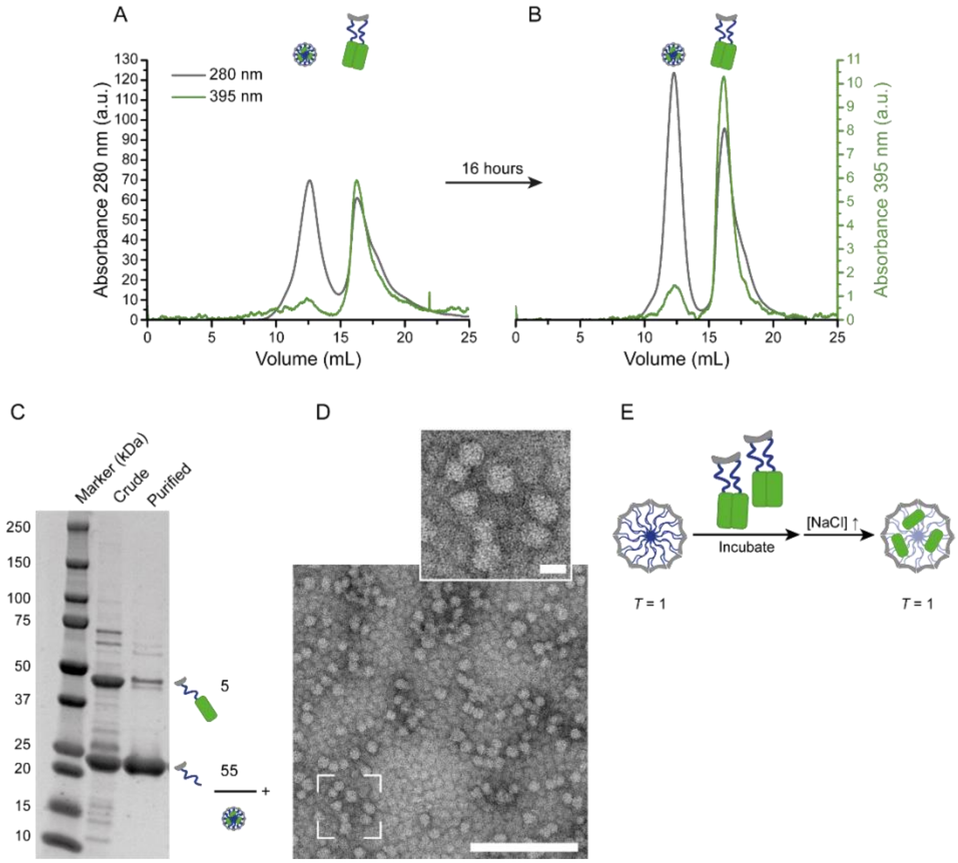

Figure S12. Analysis of mEGFP-labeling of *T*=1 VW1-VW8 ELP-CCMV capsids by mixing empty *T*=1 capsids with labeled mEGFP-VW1-VW8 ELP-CCMV dimers. A-B) SEC chromatograms of 25 µM mEGFP-VW1-VW8 ELP-CCMV and 100 µM VW1-VW8 ELP-CCMV capsid mixtures in pH 7.5. 500 mM NaCl buffer with protein absorbance measured at 280 nm (gray curve and axis) and mEGFP absorbance measured at 395 nm (green curve and axis). Mixtures were allowed to mix at 4 °C, pH 7.5, 500 mM NaCl for 90 minutes (A) or overnight (B). The increase in mEGFP signal indicates that the mEGFP-labeled dimers exchange with non-labeled dimers over time. C) SDS-PAGE analysis of SEC purification after overnight exchange. The gel was stained with Coomassie Brilliant Blue. ImageJ quantification is depicted next to the gel. D) Uranyl acetate-stained TEM micrograph of SEC-purified capsids. The area indicated with brackets is displayed as a zoomed image. Scale bars correspond to 200 nm (main image) and 20 nm (zoomed image). E) Schematic of the process we studied: empty *T*=1 capsids are mixed with mEGFP-VW1-VW8 ELP-CCMV dimers, which upon an increase in NaCl concentration exchange with non-labelled dimers to form labeled *T*=1 capsids.

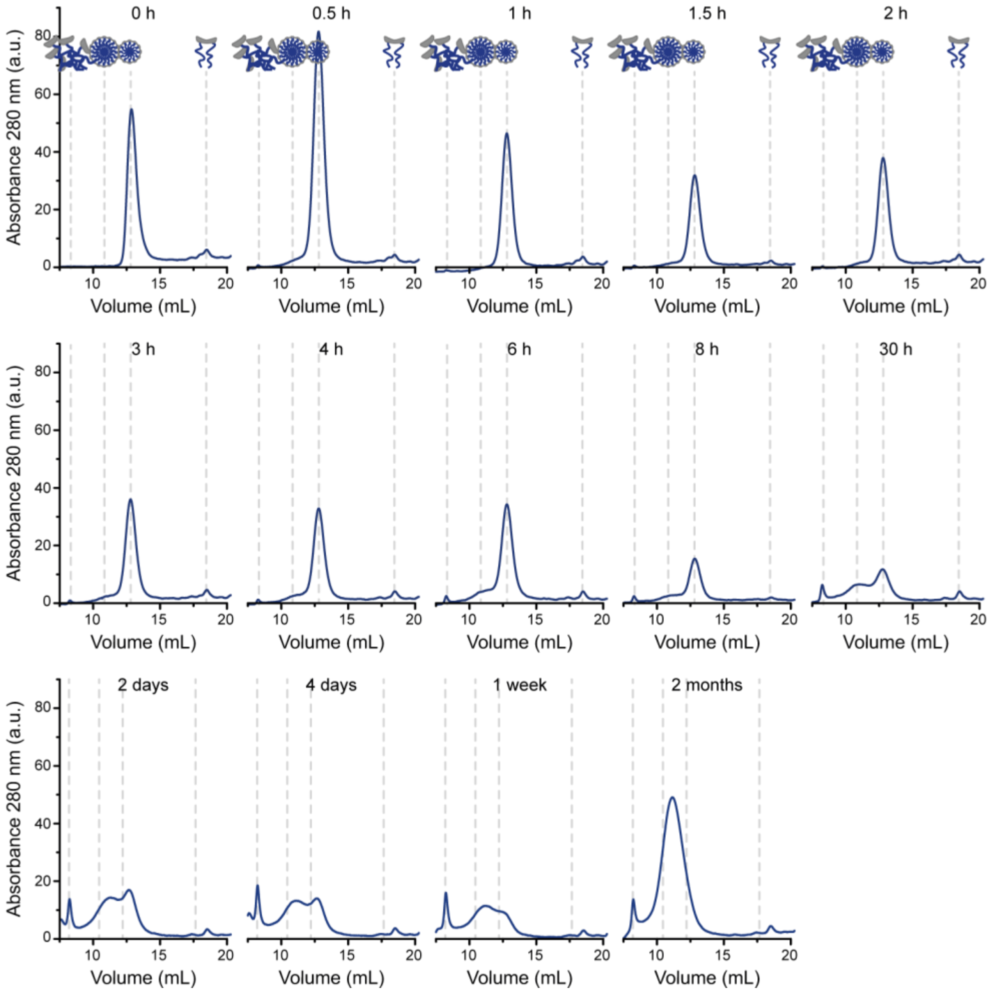

Figure S13. SEC analysis of the shift from T = 1 to T = 3 capsids (dataset 1). SEC chromatograms were measured after indicated dialysis times to pH 5.0 buffer with 500 mM NaCl at 4 °C. The results from two separate experiments are combined: 0 to 30 h and 2 days to 2 months.

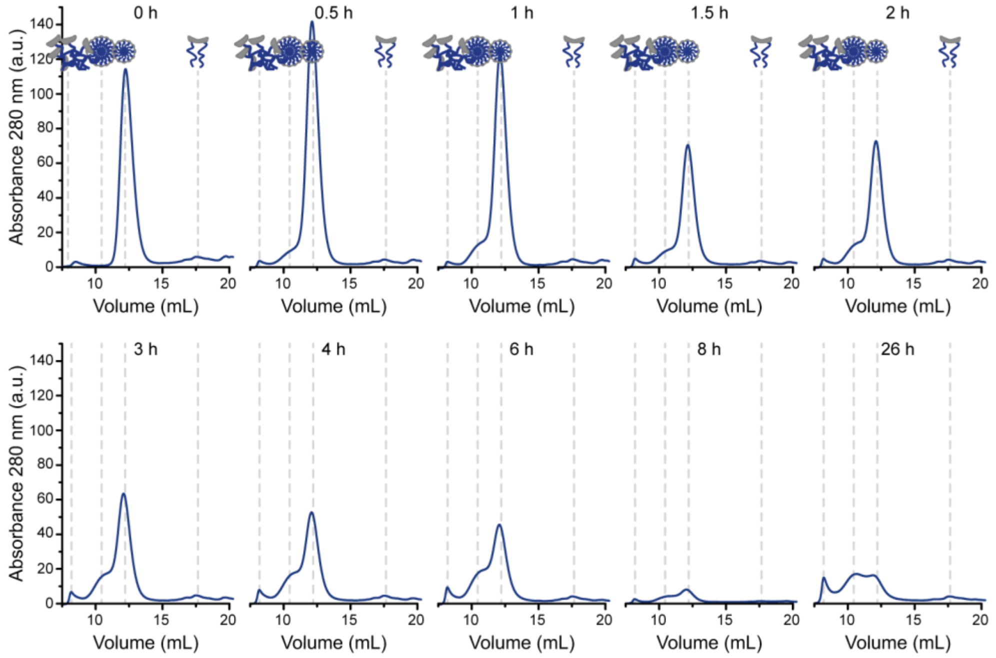

Figure S14. SEC analysis of the shift from T = 1 to T = 3 capsids (dataset 2). SEC chromatograms were measured after indicated dialysis times to pH 5.0 buffer with 500 mM NaCl at 4 °C.

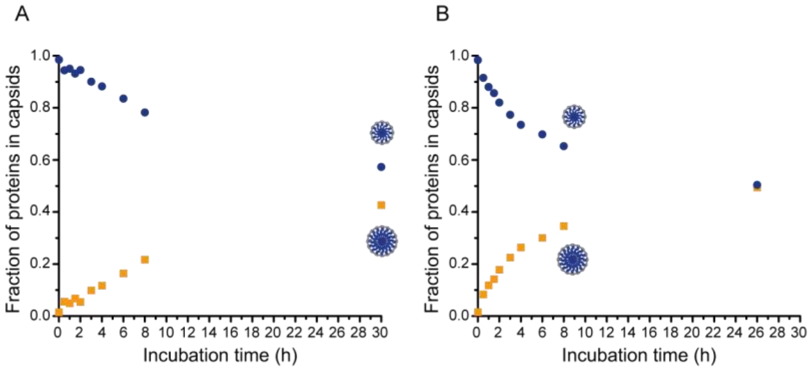

Figure S15. Comparison of time curves based on both datasets for the shift from T = 1 to T = 3 capsids. Protein fractions as T = 1 (blue circles) and T = 3 (yellow squares) capsids as determined by integration of the SEC chromatograms of Figure S11 (A) and Figure S12 (B).

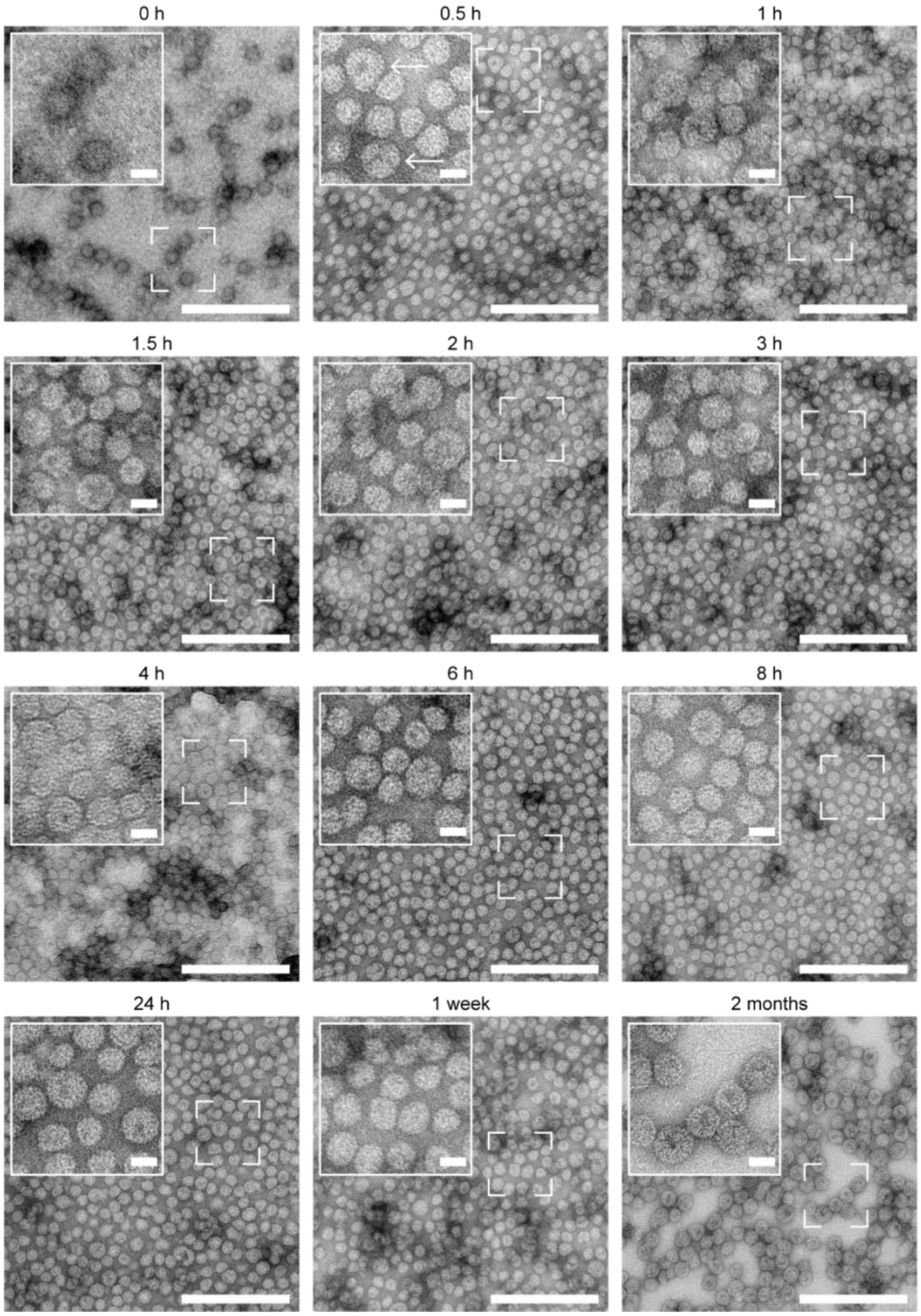

Figure S16. TEM analysis of VW1-VW8 ELP-CCMV capsids during size increase in pH 5.0 buffer. Samples were taken after the indicated dialysis times at 4 °C and negatively stained with uranyl acetate. Areas indicated with brackets are displayed as zoomed images with $T = 3$ capsids indicated by arrows. Scale bars correspond to 200 nm (main images) and 20 nm (zoomed images).

5 References

(1) Gill, S. C.; von Hippel, P. H. Calculation of Protein Extinction Coefficients from Amino Acid Sequence Data. *Anal. Biochem.* **1989**, *182* (2), 319–326.

https://doi.org/10.1016/0003-2697(89)90602-7.
(2) Schoonen, L.; Maas, R. J. M.; Nolte, R. J. M.; van Hest, J. C. M. Expansion of the Assembly of Cowpea Chlorotic Mottle Virus towards Non-Native and Physiological Conditions. *Honor Profr. Ben Feringa 2016 Tetrahedron Prize Creat. Org. Chem. Dyn. Funct. Mol. Syst.* **2017**, *73* (33), 4968–4971. https://doi.org/10.1016/j.tet.2017.04.038.
(3) van Eldijk, M. B.; Wang, J. C.-Y.; Minten, I. J.; Li, C.; Zlotnick, A.; Nolte, R. J. M.; Cornelissen, J. J. L. M.; van Hest, J. C. M. Designing Two Self-Assembly Mechanisms into One Viral Capsid Protein. *J. Am. Chem. Soc.* **2012**, *134* (45), 18506–18509. https://doi.org/10.1021/ja308132z.
(4) Schoonen, L.; Pille, J.; Borrmann, A.; Nolte, R. J. M.; van Hest, J. C. M. Sortase A-Mediated N-Terminal Modification of Cowpea Chlorotic Mottle Virus for Highly Efficient Cargo Loading. *Bioconjug. Chem.* **2015**, *26* (12), 2429–2434. https://doi.org/10.1021/acs.bioconjchem.5b00485.
(5) Timmermans, S. B. P. E. Artificial Organelles Based on Hybrid Protein Nanoparticles. PhD Thesis, Chemical Engineering and Chemistry, 2022.
(6) Minten, I. J.; Wilke, K. D. M.; Hendriks, L. J. A.; van Hest, J. C. M.; Nolte, R. J. M.; Cornelissen, J. J. L. M. Metal-Ion-Induced Formation and Stabilization of Protein Cages Based on the Cowpea Chlorotic Mottle Virus. *Small Weinh. Bergstr. Ger.* **2011**, *7* (7), 911–919. https://doi.org/10.1002/smll.201001777.
(7) Bruinsma, R. F.; Wuite, G. J. L.; Roos, W. H. Physics of Viral Dynamics. *Nat. Rev. Phys.* **2021**, 1–16. https://doi.org/10.1038/s42254-020-00267-1.
(8) Michaels, T. C. T.; Bellaiche, M. M. J.; Hagan, M. F.; Knowles, T. P. J. Kinetic Constraints on Self-Assembly into Closed Supramolecular Structures. *Sci. Rep.* **2017**, *7* (1), 12295. https://doi.org/10.1038/s41598-017-12528-8.
(9) Zandi, R.; Dragnea, B.; Travesset, A.; Podgornik, R. On Virus Growth and Form. *Phys. Rep.* **2020**, *847* (C). https://doi.org/10.1016/j.physrep.2019.12.005.
(10) Zandi, R.; Schoot, P. van der; Reguera, D.; Kegel, W.; Reiss, H. Classical Nucleation Theory of Virus Capsids. *Biophys. J.* **2006**, *90* (6), 1939–1948. https://doi.org/10.1529/biophysj.105.072975.
(11) Harms, Z. D.; Selzer, L.; Zlotnick, A.; Jacobson, S. C. Monitoring Assembly of Virus Capsids with Nanofluidic Devices. *ACS Nano* **2015**, *9* (9), 9087–9096. https://doi.org/10.1021/acsnano.5b03231.
(12) Moerman, P.; van der Schoot, P.; Kegel, W. Kinetics versus Thermodynamics in Virus Capsid Polymorphism. *J. Phys. Chem. B* **2016**, *120* (26), 6003–6009. https://doi.org/10.1021/acs.jpcb.6b01953.
(13) Zlotnick, A. To Build a Virus Capsid: An Equilibrium Model of the Self Assembly of Polyhedral Protein Complexes. *J. Mol. Biol.* **1994**, *241* (1), 59–67. https://doi.org/10.1006/jmbi.1994.1473.
(14) Keef, T.; Micheletti, C.; Twarock, R. Master Equation Approach to the Assembly of Viral Capsids. *J. Theor. Biol.* **2006**, *242* (3), 713–721. https://doi.org/10.1016/j.jtbi.2006.04.023.
(15) Rapaport, D. C. Role of Reversibility in Viral Capsid Growth: A Paradigm for Self-Assembly. *Phys. Rev. Lett.* **2008**, *101* (18), 186101. https://doi.org/10.1103/PhysRevLett.101.186101.
(16) Berthet-Colominas, C.; Cuillel, M.; Koch, M. H. J.; Vachette, P.; Jacrot, B. Kinetic Study of the Self-Assembly of Brome Mosaic Virus Capsid. *Eur. Biophys. J.* **1987**, *15* (3), 159–168. https://doi.org/10.1007/BF00263680.
(17) Asor, R.; Schlicksup, C. J.; Zhao, Z.; Zlotnick, A.; Raviv, U. Rapidly Forming Early Intermediate Structures Dictate the Pathway of Capsid Assembly. *J. Am. Chem. Soc.* **2020**, *142* (17), 7868–7882. https://doi.org/10.1021/jacs.0c01092.
(18) Zandi, R.; van der Schoot, P. Size Regulation of Ss-RNA Viruses. *Biophys. J.* **2009**, *96* (1), 9–20. https://doi.org/10.1529/biophysj.108.137489.

(19) Luque, A.; Reguera, D.; Morozov, A.; Rudnick, J.; Bruinsma, R. Physics of Shell Assembly: Line Tension, Hole Implosion, and Closure Catastrophe. *J. Chem. Phys.* **2012**, *136* (18), 184507. https://doi.org/10.1063/1.4712304.
(20) Kashchiev, D. In *Nucleation*; Elsevier, 2000; pp 113–290.
(21) Luque Santolaria, A. *Structure, Mechanical Properties, and Self-Assembly of Viral Capsids*; Universitat de Barcelona, 2011.
